# MethyNano: supervised contrastive pretraining enables robust and generalizable methylation detection from nanopore sequencing

**DOI:** 10.1093/bioinformatics/btag348

**Published:** 2026-05-28

**Authors:** Jiahui Yan, Yujie Chen, Yucong Gong, Cheng Zhang, Jing Yang

**Affiliations:** School of Control and Computer Engineering, North China Electric Power University, Beijing 102206, China; School of Control and Computer Engineering, North China Electric Power University, Beijing 102206, China; School of Computer Science, Key Laboratory of High Confidence Software Technologies, Peking University, Beijing 100871, China; School of Computer Science, Key Laboratory of High Confidence Software Technologies, Peking University, Beijing 100871, China; School of Control and Computer Engineering, North China Electric Power University, Beijing 102206, China

## Abstract

**Motivation:**

5-Methylcytosine (5mC) plays an important role in gene regulation and development. Although nanopore sequencing has enabled direct detection of 5mC, existing methods still face several limitations, including poor generalization across species and sequence contexts (CpG/CHG/CHH), as well as suboptimal integration of sequence and current signals.

**Results:**

Here, we present MethyNano, a deep learning framework incorporating a contrastive learning strategy to detect 5mC from nanopore reads. By encouraging more discriminative and stable representations, the contrastive objective improves the model’s sensitivity to rare sequence contexts and reduces its prediction uncertainty in challenging regions. Across datasets from *Arabidopsis thaliana*, *Oryza sativa*, and *Homo sapiens*, our model achieves superior performance on key metrics compared with other existing methods. Extensive cross-species and cross-motif experiments demonstrate the robust generalization performance of MethyNano, while dimensionality-reduction visualizations of learned features provide an intuitive view of the model’s efficient representation capability. Moreover, our ablation studies show that MethyNano’s architecture enables more effective integration of critical features, leading to higher predictive accuracy.

**Availability and implementation:**

The project code is available at https://github.com/baigeHUI/MethyNano and https://doi.org/10.5281/zenodo.19858400.

## 1 Introduction

5-Methylcytosine (5mC) is generated by DNA methyltransferases that transfer a methyl group to the C5 position of cytosine (Arand *et al.* 2012). As a pervasive and stable epigenetic mark, 5mC participates in diverse biological processes in eukaryotes, including transcriptional regulation, maintenance of genome stability, and parental imprinting (Ehrlich and Wang 1981, Jones and Takai 2001, Arand *et al.* 2012, Jones 2012). Beyond its biological roles, programmable methylation patterns can also serve as a durable information carrier. When coupled with DNA self-assembly, methyltransferase-mediated reactions enable efficient parallel encoding of digital bits into DNA (Zhang *et al.* 2024). Therefore, accurate single-base–resolution mapping of 5mC has broad relevance across diverse fields. Conventional methods for 5mC detection include bisulfite sequencing, TET assisted pyridine borane sequencing (TAPS), and enzymatic methyl sequencing (EM-seq). For instance, bisulfite sequencing relies on the conversion of unmodified cytosine (C) to uracil (U) by sodium bisulfite, whereas 5mC remains unconverted. During PCR amplification, uracil is read as thymine, resulting in C to T substitutions at originally unmethylated positions. Comparing the resulting sequences with the reference allows positions that remain as C to be distinguished from those converted to T, thereby discriminating 5mC from unmodified cytosine at single-base resolution (Jones and Takai 2001). These protocols provide precise localization of methylated sites, yet their reliance on chemical or enzymatic processing can compromise DNA integrity.

Nanopore sequencing enables direct analysis of DNA and RNA molecules without amplification or chemical conversion, including those carrying chemical modifications (Flusberg *et al.* 2010, Wallace *et al.* 2010, Laszlo *et al.* 2013, Rand *et al.* 2017, Garalde *et al.* 2018). During nanopore sequencing, nucleotides generate characteristic changes in ionic current as they translocate through the pore, and analysis of these signals enables inference of the underlying sequence (Wan *et al.* 2022). This platform simplifies library preparation and supports long reads with rapid sequencing throughput (Simpson *et al.* 2017, Xu and Seki 2020). Importantly, chemical modifications also perturb the raw current signal, allowing direct detection of modifications at the single-molecule level. Early nanopore modification callers were primarily based on statistical modeling coupled with alignment of signals to the reference. Nanopolish (Simpson *et al.* 2017) aligns raw current signals to the reference genome and applies a hidden Markov model (HMM) to compare signal distributions under modified and unmodified hypotheses, yielding site-level likelihood ratios for DNA methylation calling. Tombo (Stoiber *et al.* 2017), developed by ONT, performs resquiggling and offers both motif-guided model detection and reference-based comparative analyses to support diverse experimental settings and modification types. SignalAlign (Rand *et al.* 2017) uses a Bayesian belief network to integrate aligned current signals with local sequence context at candidate sites and outputs posterior methylation probabilities. As deep learning-based methods have consistently improved performance in modification detection, an increasing number of groups have turned to neural approaches. mCaller (McIntyre *et al.* 2019) trained a neural network for m6A detection. DeepSignal (Ni *et al.* 2019) combines convolutional networks with bidirectional long short-term memory networks to learn current signatures of 5mC and 6 mA directly from raw signals and outputs site-level probability predictions. Building on DeepSignal, DeepSignal-Plant (Ni *et al.* 2021) incorporates Transformer modules to better capture plant-specific methylation patterns, improving performance in complex plant genomes. In addition, DeepMP (Bonet *et al.* 2022) integrates basecalling error profiles with current signals and performs methylation prediction using a supervised Bayesian framework. methBERT (Zhang *et al.* 2021) adopts a BERT-style bidirectional Transformer architecture to enable more efficient parallel inference and reduce the computational burden of long sequences. METEORE (Yuen *et al.* 2021) ensembles multiple callers, integrating their outputs via voting and probability fusion to yield improved methylation predictions. remora, released by ONT, uses a decoupled neural architecture to support joint modeling of sequence and modification signals. NanoCon (Yin *et al.* 2024) constructs a Transformer and bidirectional GRU-based model, leveraging the classic pairwise contrastive objective N-pairs loss (Hadsell *et al.* 2006) to learn a methylation-aware representation space for discrimination. DeepPlant (Chen *et al.* 2025) focuses on CHH methylation detection in plants, using BiLSTM and Transformer components to improve the model’s reliability in CHH contexts. More broadly, nanopore-based analysis has expanded to a wider spectrum of DNA and RNA modifications. On the DNA side, direct detection of oxidative lesions such as 8-oxo-dG has been demonstrated (Pagès-Gallego *et al.* 2025), while signal deconvolution and recognition for non-canonical base systems have also been explored (Perez *et al.* 2025). On the RNA side, joint analyses have enabled parallel detection of m6A and pseudouridine (Ψ) (Huang *et al.* 2024, Alagna *et al.* 2025, Chan *et al.* 2025, *Zhang et al.* 2025). Related work has further explored modification prediction for m6A directly during basecalling (Cruciani *et al.* 2025), as well as construction of single-molecule, high-resolution m6A maps (Kang *et al.* 2025). Collectively, these advances underscore the continued momentum of nanopore sequencing in nucleic acid modification analysis and point to opportunities for improving the accuracy and robustness of 5mC detection.

Although methylation detection has advanced rapidly, several gaps remain. Cross-species generalization is still a major challenge: models often perform well on the species they were trained on but degrade substantially when transferred to other organisms, reflecting differences in both modification abundance and genomic distribution across species (Richards 2011). In addition, 5mC occurs in three sequence contexts (CpG, CHG, and CHH), yet reliable calling in the asymmetric CHH context remains difficult (Ni *et al.* 2021). This challenge arises from the low prevalence of CHH methylation in eukaryotic cells (Cokus *et al.* 2008, Lister *et al.* 2008, Niederhuth *et al.* 2016) and the heterogeneous, complex current patterns associated with CHH events, which are harder to model accurately (Yin *et al.* 2024). Moreover, reliable 5mC detection requires both sequence context and current signals, which should be integrated in a way that captures their cross-modal interactions. However, the widely used baseline of simple feature concatenation (Ni *et al.* 2019, Bonet *et al.* 2022) cannot model the rich nonlinear dependencies between modalities, thereby leaving sequence and signal representations only weakly coupled and ultimately constraining further improvements in performance.

In recent years, contrastive learning has emerged as a powerful paradigm in representation learning (Jia *et al.* 2026), enabling models to acquire discriminative and robust feature representations by explicitly pulling similar samples closer and pushing dissimilar ones apart within the embedding space (Hadsell *et al.* 2006). This approach is particularly well-suited for nanopore-based methylation detection, where current signal differences between methylated and unmethylated sites are subtle and prone to noise interference. Additionally, certain motifs (e.g. CHH) exhibit severe sample scarcity, rendering traditional classification losses inadequate for optimizing decision boundaries. By learning from the relative distinctions between positive and negative samples, contrastive learning enhances the model’s sensitivity to weak signals and rare motifs (Hu *et al.* 2024). Despite recent attempts to integrate contrastive learning into nanopore methylation detection (Yin *et al.* 2024), the N-pairs loss they adopted is restricted to predefined sample pairs derived from label information, failing to model structural relationships between individual samples and their broader categories, thereby limiting the model’s discriminative capability.

Here, to address these challenges, we develop MethyNano, a novel deep learning framework for accurate 5mC detection that integrates supervised contrastive pretraining with advanced multimodal architecture design. Our framework introduces three key innovations. First, MethyNano introduces the supervised contrastive learning objective SupCon (Khosla *et al.* 2020). By enabling comprehensive comparisons among all samples within a single batch, MethyNano learns a more discriminative representation space that effectively captures distinctions between positive and negative instances. Second, we design a signal encoder based on multi-head convolutional neural networks coupled with FiLM conditional modulation (Pérez *et al.* 2018). This architecture captures multi-scale patterns in raw signals and adaptively integrates global statistical features. Third, we replace simple feature concatenation with a symmetric cross-attention fusion module, which enables deep bidirectional interaction between DNA sequence embeddings and signal representations, thereby modeling their nonlinear dependencies more effectively. These improvements allow MethyNano to achieve state-of-the-art performance across multiple species, including *Arabidopsis thaliana*, *Oryza sativa* and *Homo sapiens*. It consistently outperforms existing methods such as DeepPlant, remora, rockfish, and NanoCon on key metrics including AUROC, AUPRC, and F1 score. Furthermore, MethyNano demonstrates strong generalization and robustness in cross-motif prediction, while systematic ablation studies confirm the importance of its input modalities and key architectural components. In-depth error analysis further clarifies its decision boundaries and limitations, offering a comprehensive understanding of its predictive behavior. Collectively, our work shows that MethyNano provides substantial improvements in cross-species generalization, multi-motif adaptability, and multimodal fusion, offering a more accurate and robust tool for nanopore 5mC detection.

## 2 Materials and methods

### 2.1 Datasets and preprocessing

We use publicly available datasets and apply several optimizations to the data extraction pipeline. All nanopore sequencing data used in this study were generated on the Oxford Nanopore R10.4 platform. Specifically, we adopt the *A. thaliana* and *O. sativa* datasets curated by (Bai *et al.* 2024). The corresponding nanopore and bisulfite sequencing data are available through the National Center for Biotechnology Information (NCBI) under BioProject PRJCA023349. The *A. thaliana* reference genome is obtained from NCBI (GCF_000001735.4_TAIR10.1) (Wheeler *et al.* 2008), whereas the *O. sativa* reference genome is obtained from EnsemblPlants (IRGSP-1.0) (Howe *et al.* 2020). In addition, we use a *H. sapiens* dataset from (Genner *et al.* 2025) under BioProject PRJNA646948. The matched bisulfite sequencing results and reference genome are obtained from the ENCODE project (ENCFF835NTC) (ENCODE Project Consortium 2012). For each species, we select an extensive set of high-quality reads to construct the final datasets.

**Figure 1:**
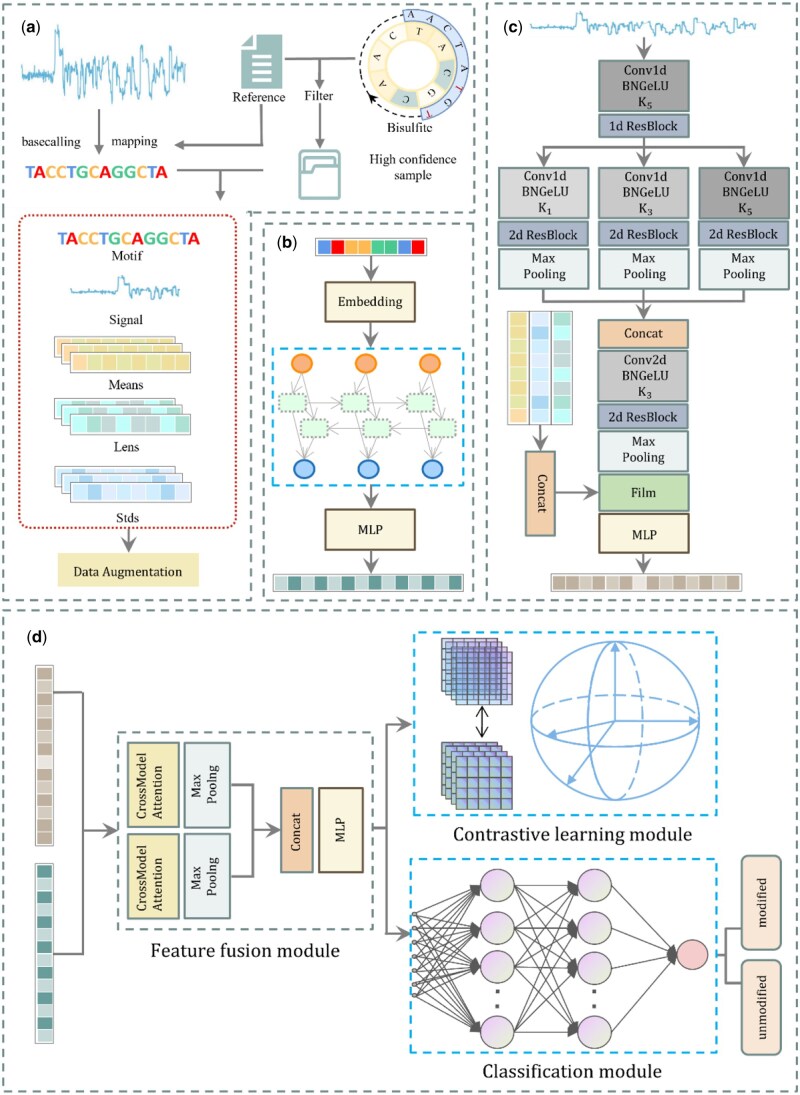
Overall architecture of MethyNano. (a) Workflow for dataset construction from sequencing data. (b–d) Schematic of the deep learning pipeline: (b) sequence feature extraction module. (c) signal feature extraction module. (d) feature fusion module with downstream task heads. We first apply data augmentation to the processed data to generate a batch of sample pairs (original signals and their augmented counterparts). Each pair is then fed into two weight-sharing branches with identical architecture to obtain feature representations, which are used for contrastive learning. After pretraining, we discard the contrastive projection head and replace it with a classification head to further fine-tune the model, yielding the final methylation predictions.

Figure 1a summarizes our data processing pipeline. Raw nanopore signals are basecalled and aligned to the reference genome using Dorado (v0.7.2) with the dna_r10.4.1_e8.2_400bps_hac@v4.2.0 model, and the resulting BAM files were sorted using Samtools (v1.21). For bisulfite sequencing (BS-seq) data, we likewise align reads to the same reference genome and identify methylated sites using Bismark (v0.24.0) (Krueger and Andrews 2011). Next, we construct candidate samples by extracting 13 bp motif windows centered on cytosines from each read. On the signal side, the raw nanopore current is aligned to the motif sequence, and the signal segment corresponding to each base is resampled to 100 points to ensure a consistent input length per position. We then compute the mean, standard deviation, and the original number of samples for each base-level segment to capture local signal statistics. Subsequently, candidate cytosines are assigned binary methylation labels using BS-seq. We retain only sites with BS-seq coverage above 9 and apply methylation-level thresholds of 0.1/0.9, 0.1/0.8, and 0.1/0.7 for CpG, CHG, and CHH respectively, ensuring high-confidence positives while maintaining a sufficient set of reliable negatives. From the processed candidates, we extract positive and negative instances to form the final dataset and perform an additional round of sampling to balance the class ratio. We then construct data records. Each record contains six elements: the 13 bp motif, the aligned raw signal, signal means, signal standard deviations, signal lengths, and the binary methylation label of the centered cytosine. Finally, records are split into training, validation, and test sets in a 7:2:1 ratio. Using this procedure, we construct 200 000 records for each of the *A. thaliana*, *O. sativa*, and *H. sapiens* datasets for model training.

### 2.2 Architecture of our model

MethyNano is a deep learning–based methylation caller with a hybrid architecture composed of multi-head 1D-CNNs, a Transformer, and a bidirectional LSTM. The model is trained jointly with contrastive and classification objectives (Tables S1–S5, available as supplementary data at *Bioinformatics* online).

**Table 1:** Performance evaluation of MethyNano, NanoCon, remora, rockfish, and DeepPlant on test sets from different species. **Bold values indicate the best performance for each metric.**

Metric	Dataset	MethyNano	NanoCon	remora	rockfish	DeepPlant
Precision	*A. thaliana*	**0.8426**	0.7445	0.7605	0.7882	0.8316
	*H. sapiens*	0.8380	0.7244	0.7631	0.8047	**0.8476**
	*O. sativa*	0.8326	0.7253	0.7606	0.7963	**0.8462**
Accuracy	*A. thaliana*	**0.8382**	0.7322	0.7607	0.8024	0.8309
	*H. sapiens*	**0.8371**	0.7363	0.7611	0.7992	0.8312
	*O. sativa*	**0.8347**	0.7403	0.7923	0.7950	0.8306
Recall	*A. thaliana*	**0.8321**	0.7037	0.7590	0.8253	0.8286
	*H. sapiens*	**0.8348**	0.7600	0.7558	0.7890	0.8067
	*O. sativa*	**0.8375**	0.7736	0.7490	0.7923	0.8075
F1 score	*A. thaliana*	**0.8366**	0.7231	0.7598	0.8063	0.8301
	*H. sapiens*	**0.8364**	0.7414	0.7595	0.7968	0.8266
	*O. sativa*	**0.8350**	0.7482	0.7548	0.7943	0.8264
AUROC	*A. thaliana*	**0.9202**	0.8218	0.8444	0.8883	0.9149
	*H. sapiens*	**0.9196**	0.8220	0.8439	0.8839	0.9148
	*O. sativa*	**0.9173**	0.8265	0.8391	0.8814	0.9152
AUPRC	*A. thaliana*	**0.9215**	0.8243	0.8437	0.8873	0.9165
	*H. sapiens*	**0.9204**	0.8239	0.8432	0.8814	0.9159
	*O. sativa*	**0.9183**	0.8300	0.8395	0.8800	0.9177

**Table 2:** Results of the ablation experiments for MethyNano. Bold values indicate the best performance for each metric.

model	Accuracy	Precision	F1 score	Recall	AUROC	AUPRC
MethyNano	**0.8382**	**0.8426**	**0.8366**	0.8321	0.9202	**0.9215**
mask_sequence	0.7604	0.7492	0.7647	0.7807	0.8474	0.8492
mask_signal	0.8235	0.8124	0.8260	**0.8399**	0.9086	0.9101
mask_means	0.7578	0.7509	0.7601	0.7696	0.8378	0.8397
mask_stds	0.8259	0.8216	0.8264	0.8313	0.9081	0.9083
mask_lens	0.8373	0.8421	0.8359	0.8318	**0.9203**	0.9209
mask_multiCNN	0.8297	0.8299	0.8291	0.8282	0.9129	0.9139
mask_fuser	0.8252	0.8232	0.8250	0.8269	0.9079	0.9096
mask_contrastive	0.8262	0.8301	0.8271	0.8317	0.9091	0.9073

The overall framework is organized into five modules: data preprocessing module, feature extraction module, feature fusion module, contrastive learning module and classification module. In the data preprocessing module, we apply data augmentation to the signal of each record and recompute the corresponding statistical features to generate an augmented record, which is paired with the original record as a sample pair. The augmented sample introduces localized perturbations while largely preserving the overall waveform of the original signal (Fig. S1, available as supplementary data at *Bioinformatics* online). The feature extraction module learns sequence features and signal features in parallel, then passes the resulting tokens to the feature fusion module for cross-modal integration and interaction. The contrastive learning module computes the contrastive objective, encouraging representations from the same class to be closer while pushing different classes apart. The classification module uses the learned features to predict the methylation state of the input sequence.

### 2.3 Feature extraction module

The feature extraction module learns representations from motif sequences and current signals through two parallel branches. The input sequences are one-hot encoded and projected into an embedding space suitable for the network. A Bi-LSTM then extracts sequence features from the resulting embeddings. The current signals are first processed with residual blocks (He *et al.* 2016). The resulting features are fed into a multi-head CNN to capture signal patterns at multiple scales. To improve the model’s adaptability to sample-level variation in global signal statistics, we apply FiLM conditioning (Pérez *et al.* 2018) to the convolutional features in the signal branch. Specifically, we form a statistical vector z by concatenating signal means, signal standard deviations, and signal lengths. A lightweight MLP maps z to channel-wise scaling and shifting parameters γ and β:


(1) $$(\gamma ,\beta )=f(z),\;\gamma ,\beta \in {R\;}^{B\times C};$$

We denote the signal representation produced by the multi-head 1D CNN as $X\in R^{B\times C\times T}$, where B is batch size, C is the number of channels, and T is the number of time steps. FiLM performs a channel-wise affine transformation and broadcasts it along the temporal dimension:


(2) $$Y_{\mathrm{out}}=\gamma ⊙X+\beta ;$$

where ⊙ denotes element-wise multiplication. This design uses signal statistics to modulate channel activations, leading to more stable and better separated representations for downstream prediction.

### 2.4 Feature fusion module

The feature fusion module forms the core of MethyNano. It is designed to tightly couple sequence information with signal features produced by the feature extraction module. We achieve this coupling with symmetric cross-modal attention (Vaswani *et al.* 2017). Specifically, we compute multi-head attention in both directions. In one direction, sequence features are used as queries, while signal features serve as keys and values. In the other direction, signal features act as queries, with sequence features providing keys and values. The resulting attention outputs are passed through an FFN followed by normalization, then concatenated to form the fused representation. The core computation is summarized in Equations (3)–(6).

For notation, we denote motif tokens as $S\in R^{L_{s}\times d}$ and signal tokens as $G\in R^{L_{G}\times d}$, yielding:


(3) $$\mathrm{Attn}(Q,K,V)=\mathrm{softmax}(\frac{QK^{T}}{\sqrt{d_{k}}})V;$$

(4) $$\mathrm{MHA}(X,Y)=\mathrm{Concat}{\left(\mathrm{Attn}(XW_{Q,i},YW_{K,i},YW_{V,i})\right)}_{i=1}^{h}W_{O};$$

(5) $${\tilde{S}}^{\left(1\right)}=\mathrm{MHA}(S,G),{\tilde{G}}^{\left(1\right)}=\mathrm{MHA}(G,S);$$

(6) $${\tilde{S}}^{\left(2\right)}=\mathrm{MHA}({\tilde{S}}^{\left(1\right)},{\tilde{G}}^{\left(1\right)}),{\tilde{G}}^{\left(2\right)}=\mathrm{MHA}({\tilde{G}}^{\left(1\right)},{\tilde{S}}^{\left(1\right)});$$

Here, $S\in R^{L_{s}\times d}$ and $G\in R^{L_{G}\times d}$ denote the token matrices from the sequence and signal branches, respectively.$\;L_{S}$ and $L_{G}$ specify the token lengths, and d denotes the embedding dimension. In our design, the sequence branch uses 13-mer motifs, so $L_{S}=13$. In the signal branch, raw current signals are aligned to the corresponding motif sequences and summarized at base level. This yields one signal token per base across the same 13 positions. Equation (3) gives the standard form of single head attention. It scores the compatibility between Q and K, applies scaling followed by softmax normalization to obtain attention weights, then uses these weights to aggregate V. Equation (4) defines multi head attention. Attention is computed in parallel across h subspaces, where head i uses its own linear projections $W_{Q,i},W_{K,i},W_{V,i}$. The outputs from all heads are concatenated along the channel dimension, then mapped back to the shared dimension d via $W_{O}$. In our cross-modal setting, (X,Y)=(S,G) corresponds to sequence to signal attention, whereas (X,Y)=(G,S) corresponds to signal to sequence attention.

### 2.5 Contrastive learning module

To encourage a well-structured metric space for fused representations, we introduce a contrastive objective during training and pair each sample with a current-augmented view. The original record and its augmented counterpart are fed through the network to produce two fused embeddings $h_{i}$ and $h_{i}^{+}$. We start from the standard InfoNCE formulation (van den Oord *et al.* 2018). For contrastive learning, $h_{i}$ serves as the anchor, $h_{i}^{+}$ is treated as the positive, and all other examples in the same batch, together with their augmented views, form the negative set. The fused embeddings are further transformed by a two-layer MLP projection head g(⋅) to obtain lower-dimensional vectors. These projected vectors are L2 normalized onto the unit hypersphere. Cosine similarity is used to measure distances between them:


(7) $$z_{i}=\frac{g(h_{i})}{{\left|\left|\;g(h_{i})\right|\right|}_{2}\;},\;z_{i}^{+}=\frac{g(h_{i}^{+})}{{\left|\left|g(h_{i}^{+})\right|\right|}_{2}};$$

(8) $$s(u,v)=u^{T}v;$$

To better shape the representation space by pulling same-class samples closer while pushing different classes apart, we unify the instance-level strong pairing in InfoNCE with the within-class aggregation in supervised contrastive learning SupCon (Khosla *et al.* 2020). For a batch where two views are concatenated, we obtain an anchor index set {1,…, 2B}. For each anchor i, we define a strong positive set $P_{i}^{\mathrm{pair}}\;$which contains only the paired instance from the other view, and a weak positive set $P_{i}^{\mathrm{weak}}=\{j\neq i:y_{j}=y_{i},j\notin P_{i}^{\mathrm{pair}}\}$ which includes same-class but non-paired samples. We denote the temperature parameter by τ>0, and use α∈[0,1) to weight weak same-class pairs. The weighted supervised contrastive term for anchor i is defined as:


(9)  ℓi=-1|Pipair|+α|Piweak|[∑j∈Pipairlog⁡exp⁡(s(zi,zj)/τ)∑k≠iexp⁡(s(zi,zk)/τ)+α∑j∈Piweaklog⁡exp⁡(s(zi,zj)/τ)∑k≠iexp⁡(s(zi,zk)/τ)];

where k∈{1,…, 2B} with k≠i indexes candidate contrasts in the batch, and s(⋅,⋅) denotes cosine similarity. Averaging over the 2B anchors yields the contrastive loss:


(10) $$L_{\mathrm{con}}=\frac{1}{2B}\sum_{i=1}^{2B}\ell_{i};\;$$

When α=0, the objective reduces to standard InfoNCE and aligns only the strong paired positives $\left(z_{i},z_{i}^{+}\right)$. When α>0, the weak same-class term introduces a lightly weighted SupCon constraint. It encourages within-class directional consistency while maintaining clear separation from other classes (Fig. S7, available as supplementary data at *Bioinformatics* online). In this work, we set τ=0.1 and α=0.01.

### 2.6 Classification module

The classification module is used to predict the class of each input sample. It shares the same backbone network with the contrastive projection head. Unlike the projection head that applies L2 normalization, the classification module outputs logits. We train the classifier with cross-entropy loss. This objective measures the mismatch between the predicted class-probability distribution and the ground-truth label, and is defined as:


(11) $${\;L}_{\text{cls}}=-\frac{1}{N}\sum_{i=1}^{N}y_{i}\log p_{i}+(1-y_{i})\log\left(1-p_{i}\right);$$

Here, $y_{i}\in \{0,1\}$ denotes the ground-truth label, $p_{i}$denotes the predicted probability for sample i, and N denotes the batch size.

To promote stable optimization and robust performance, we use a two-stage training strategy. The model is first pretrained with a contrastive objective, during which the classification head is omitted from the forward pass and does not receive gradient updates. The contrastive projection head is then replaced with a classification head for fine-tuning. Fine-tuning is performed with a smaller learning rate, and performance is evaluated on the validation set at each epoch while monitoring multiple metrics to select the best model. This training scheme supports stable convergence and enhances the model’s discriminative ability.

### 2.7 Performance metrics

Methylation prediction is fundamentally a binary classification task, and the primary evaluation metrics are defined in Equations (12)–(15):


(12) $$\mathrm{Accuracy}=\frac{\mathrm{TP}+\mathrm{TN}}{\mathrm{TP}+\mathrm{TN}+\mathrm{FP}+\mathrm{FN}};\;$$

(13) $$\mathrm{Precision}=\frac{\mathrm{TP}}{\mathrm{TP}+\mathrm{FP}};\;$$

(14) $$\mathrm{Recall}=\frac{\mathrm{TP}}{\mathrm{TP}+\mathrm{FN}};\;$$

(15) $$F1\;\mathrm{score}=\frac{2\times \mathrm{Precision}\times \mathrm{Recall}}{\mathrm{Precision}+\mathrm{Recall}}$$

Among them, accuracy measures the proportion of correctly classified instances. Precision quantifies the fraction of true positives among positive predictions, whereas recall measures the fraction of correctly identified positives among all positives. The F1 score is defined as the harmonic mean of precision and recall. In addition, to more comprehensively evaluate model performance, we report AUROC (area under the receiver operating characteristic curve) and AUPRC (area under the precision–recall curve). These metrics characterize performance from the true positive rate-false positive rate and precision-recall perspectives respectively, providing an objective view of model behavior across varying decision thresholds.

### 2.8 Training settings

We train and evaluate the model in PyTorch on an RTX 4090 GPU. We use the AdamW optimizer (weight decay = $1\times 10^{-4}$) with a linear warm-up schedule that increases the learning rate from $1\times 10^{-4}$ to $1\times 10^{-3}$ over the first five epochs to stabilize early training. Model selection is based on validation AUPRC and F1 score, which are evaluated at the end of each epoch. Predicted methylation probabilities are converted into binary predictions using a fixed decision threshold of 0.5. To reduce overfitting and avoid unnecessary checkpoints, we save only the most recent checkpoint and the best-performing checkpoint on the validation set. Regarding network design, we use pre-layer normalization and a residual-guided scheme to ease optimization in deep architectures and improve training stability. We also add dropout in each module to enhance generalization. During the contrastive learning stage, we train the model for 50 epochs, followed by 25 epochs of fine-tuning. The batch size is set to 512 throughout. During fine-tuning, the model typically reaches its best validation performance between epochs 17 and 20. These hyperparameters are refined through repeated comparisons and adjustments, aiming to ensure that the model is trained stably and reproducibly.

## 3 Results

### 3.1 Comparison results on benchmark datasets

In this study, we evaluate MethyNano on three distinct datasets: *A. thaliana*, *O. sativa*, and *H. sapiens*. Details on the data sources and dataset construction are provided in Section 2.1, Datasets and Preprocessing. We compare our model against four established methylation prediction models: NanoCon (v1.2.1), remora (v3.2.0), rockfish (v0.3), and DeepPlant (v1.1.0). To ensure a fair evaluation, we make adjustments to each model’s data loader and retrain all models on our datasets. All models are trained and tested separately for each species, without cross-species pooling. The evaluation covers cytosine-centered methylation contexts, including CpG, CHG, and CHH. In addition, to reduce discrepancies due to implementation details, we enforce consistent data splitting and metric computation across all methods. The experiments are conducted on the same computing platform to ensure that differences in computational resources do not affect the objectivity of the comparisons.

**Figure 2:**
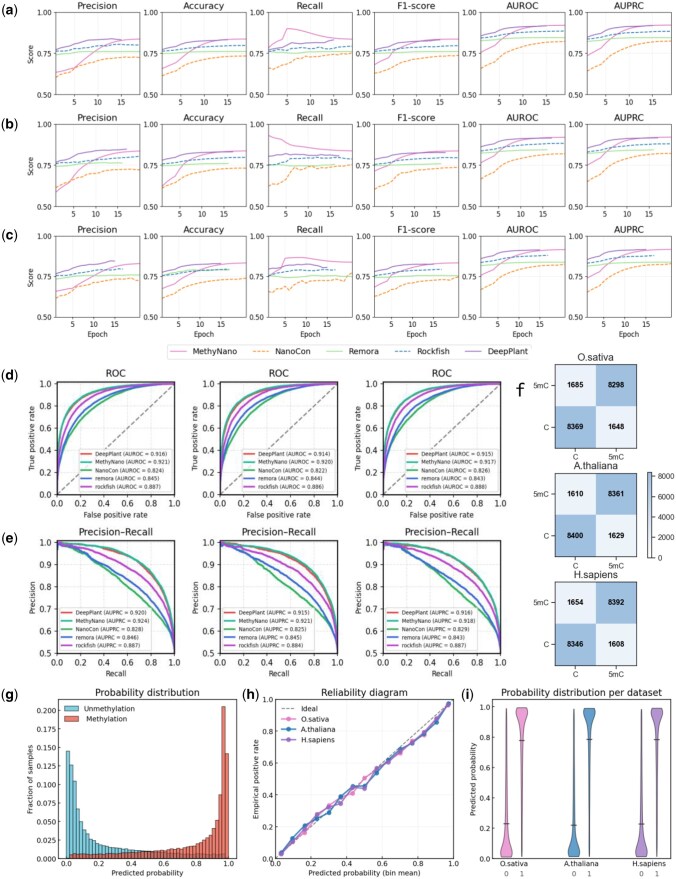
Training curves and performance evaluation of MethyNano on the constructed datasets. (a–c) Metric curves of the five models on the constructed validation sets of *A. thaliana*, *H. sapiens*, and *O. sativa* over training epochs. (d, e) ROC and PR curves of the five models on the *A. thaliana*, *H. sapiens*, and *O. sativa* test sets. (f) Classification results of MethyNano on the three species-specific test sets. (g) Overall distribution of predicted scores for methylated and unmethylated cytosines across all datasets. (h) Calibration curves comparing MethyNano’s predicted probabilities with ground-truth labels on the three species datasets. (i) Probability distributions of MethyNano’s predictions across different species datasets.

As shown in Table 1 and Fig. 2, MethyNano achieves the best results on the vast majority of metrics across the three datasets. Specifically, on the *A. thaliana* dataset, MethyNano reaches 0.8426 in precision, 0.8382 in accuracy, 0.8321 in recall, 0.8366 in F1 score, 0.9202 in AUROC, and 0.9215 in AUPRC, outperforming the other four models on all metrics. On the *H. sapiens* and *O. sativa* datasets, MethyNano is slightly lower than DeepPlant in precision, while it remains leading on the other metrics. In addition, the performance differences across datasets suggest that interspecies variations in signal distributions influence model performance. For example, all models generally achieve higher AUROC and AUPRC on the *A. thaliana* dataset, and the performance gaps between models are also more pronounced, which may be attributed to clearer separability between methylated and unmethylated signals in *A. thaliana*. In contrast, signal patterns in *H. sapiens* may be more complex, with higher levels of noise and variability, thereby amplifying stability differences among model architectures. Nevertheless, MethyNano maintains clear advantages on multiple challenging datasets, supporting the effectiveness and robustness of our approach.

### 3.2 Cross-species prediction

In practical application scenarios, 5mC detection models are typically trained using labeled data from only a limited number of species. However, during deployment, they must handle samples from different species and diverse backgrounds. Therefore, beyond the within-species benchmark evaluation, we further investigate MethyNano’s predictive performance under cross-species settings. Specifically, after obtaining the best-performing checkpoint on the *A. thaliana* training and validation sets, we directly transfer MethyNano to the test sets of all three species for inference and evaluation. To ensure reliable conclusions, we use NanoCon, remora, rockfish, and DeepPlant as baselines and follow the same training and testing protocol as MethyNano.

**Figure 3:**
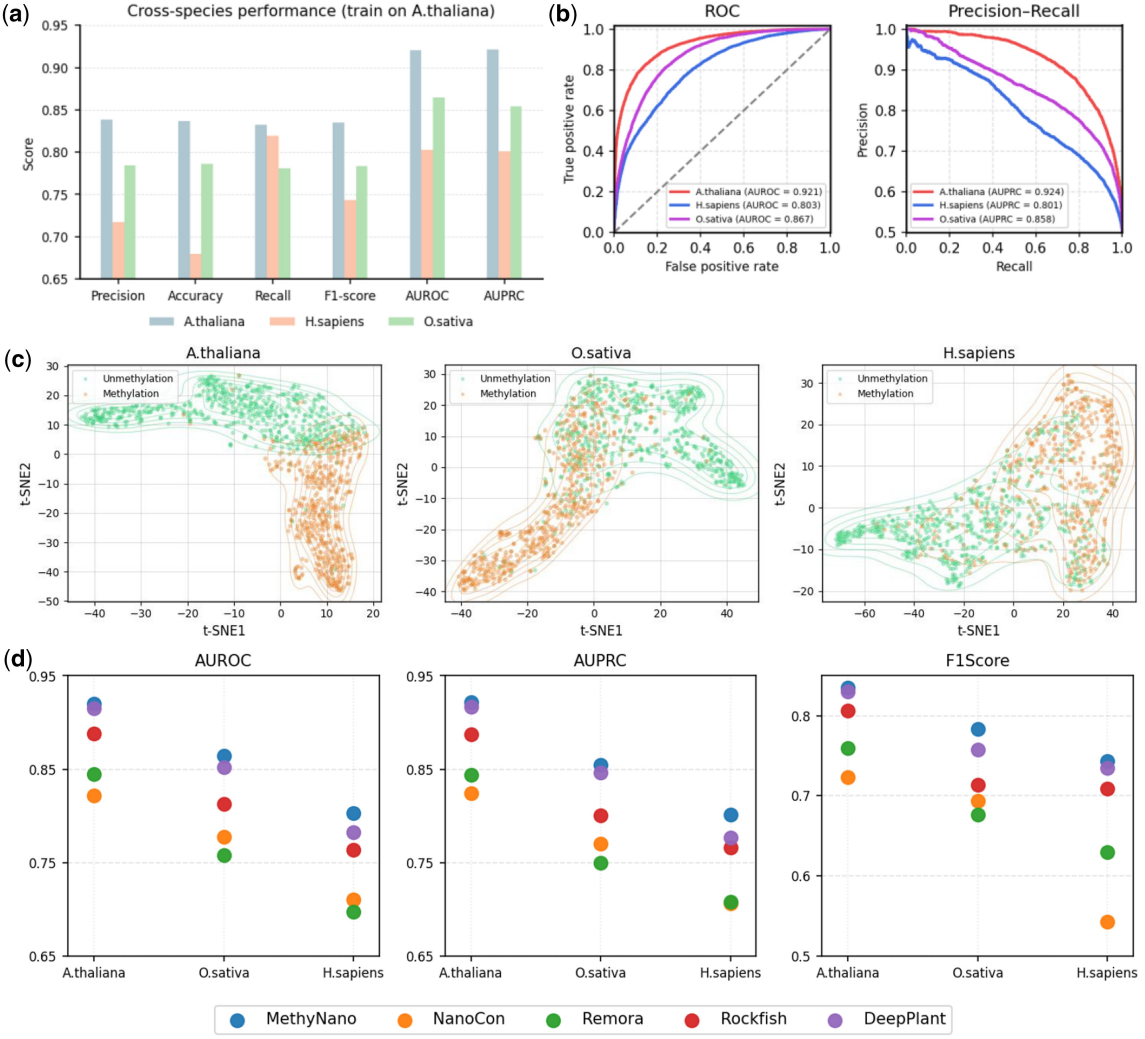
Cross-species prediction results of MethyNano trained on the *A. thaliana* dataset. (a) Metric evaluation of the model on three test sets. (b) ROC and PR curves on the test sets from three species. (c) t-SNE visualization of features extracted by the model from different species test sets. (d) Cross-species performance comparison between MethyNano and other models.

As shown in Fig. 3, we train MethyNano on the *A. thaliana* dataset and then evaluate it on the test sets of all three species. Figure 3a shows a pronounced performance drop on the *O. sativa* and *H. sapiens* test sets across all metrics. This pattern indicates that although the model still retains a degree of sensitivity, its overall discriminative ability and prediction confidence deteriorate under cross-species transfer. The degradation becomes more severe when the target domain shifts from plant to human data. To analyze the factors contributing to the observed cross-species performance degradation, we quantified the sample sizes of CpG, CHG, and CHH contexts in each species-specific dataset (Table S6, available as supplementary data at *Bioinformatics* online). We also examined the five most frequent motifs in the data of each species (Fig. S2, available as supplementary data at *Bioinformatics* online). The results show that both the proportions of CpG, CHG, and CHH samples and the most frequent motifs differ across species. These differences may partly explain the reduced cross-species performance, as variation in sequence composition can reshape the signal patterns observed by the model and reduce the transferability of learned representations across species. To further investigate the factors underlying the reduced cross-species performance, we visualize the features extracted by MethyNano from three species using t-SNE (Fig. 3c). We observe that in the *A. thaliana* test set, unmethylated and methylated samples form a relatively clear separation structure, whereas in the other two datasets the corresponding clusters appear more diffuse and less structured. This observation further suggests that cross-species distribution shifts reduce the separability of the learned representations. Nevertheless, MethyNano still outperforms the other four models in cross-species testing (Fig. S3d and Fig. S3, available as supplementary data at *Bioinformatics* online), indicating stronger cross-species generalization. Similar trends are also observed when models are trained on the *O. sativa* and *H. sapiens* datasets (Fig. S4, available as supplementary data at *Bioinformatics* online, Supplementary Data 4, available as supplementary data at *Bioinformatics* online). Although some performance degradation is observed, MethyNano continues to provide informative predictions across species, highlighting its potential and practical utility for complex biological data.

**Figure 4:**
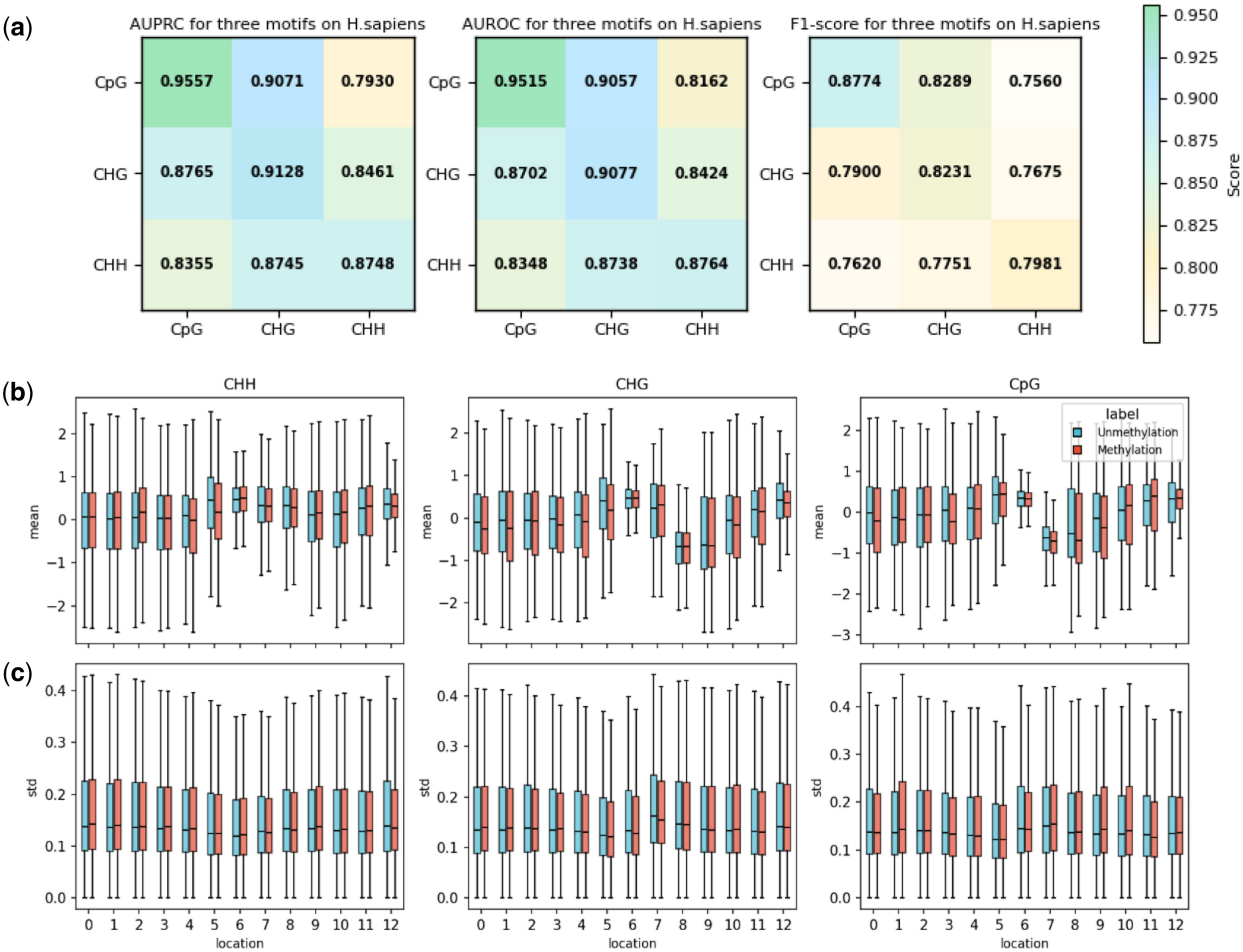
Model evaluation results and feature distribution analysis across different methylation motif contexts. (a) Heatmaps of AUPRC, AUROC, and F1 score for all train–test combinations across CpG, CHG, and CHH motifs. (b, c) Distributions of signal statistics across three motif-specific test sets: (b) distribution of signal means; (c) distribution of signal standard deviations.

### 3.3 Evaluation of 5mC in three motif contexts

After comprehensively analyzing MethyNano’s performance across species, we further assess its robustness from the perspective of sequence context by evaluating its predictive capability across three motif types (CpG, CHG, and CHH; H = A, C, or T). As 5mC patterns can vary substantially across motif contexts, we partition the *H. sapiens* dataset into CpG, CHG and CHH subsets to assess MethyNano’s performance and generalization under different sequence backgrounds.

As shown in Fig. 4a, MethyNano’s transferability varies markedly across the three motif contexts. Overall, performance is highest when training and testing are conducted within the same motif context, whereas cross-motif transfer leads to varying degrees of degradation. In particular, training on CpG and testing on CHH leads to a pronounced drop in both AUPRC and F1 score. In comparison, training on CHG yields relatively stable performance across all three motif-specific test sets, suggesting that CHG-associated signal patterns are inherently more transferable across motif contexts.

We further validate this observation by repeating the experiments with NanoCon, remora, rockfish, and DeepPlant as baselines under the same data-splitting and evaluation protocol, which helps control for method-specific effects. Figure S5, available as supplementary data at *Bioinformatics* online summarizes the cross-motif evaluation results, reporting six metrics (Accuracy, Precision, Recall, F1 score, AUROC, and AUPRC) for all methods. Overall, cross-motif transfer generally degrades performance in line with Fig. 4a, and the extent of degradation differs across models. MethyNano remains the most robust under cross-motif deployment, showing particularly stable performance in CHG-related transfer settings (Fig. S5b, available as supplementary data at *Bioinformatics* online; Supplementary Data 3, available as supplementary data at *Bioinformatics* online).

To explore the potential relationship between signal variations and cross-motif transfer performance, we plot the distributions of signal statistics for the motif-specific test sets (Fig. 4b and c). We find that the position-wise distribution patterns of both current means and standard deviations differ across motif contexts. Among the three motifs, CHH shows the most pronounced fluctuations, with a clear shift in the mean distribution near the central position. In contrast, CpG displays more compact distributions for both mean and standard deviation, along with more stable methylation-associated differences. This trend is consistent with the stronger separability of CpG observed in Fig. 4a.

### 3.4 Ablation study

Although the preceding experiments demonstrate the robust 5mC detection capability of MethyNano, the specific contributions of individual components still require clarification. Therefore, we conduct a series of ablation experiments to quantitatively assess the impact of both input features and architectural choices. On the input side, we separately mask the DNA sequence, the per-base raw signal, and the signal statistics (means, standard deviations, and lengths) during training. On the architectural side, we evaluate three model variants: (i) replacing the multi-head CNN in the signal feature extractor with a conventional CNN; (ii) substituting the feature fusion module with simple vector concatenation; and (iii) completely removing the contrastive learning module. This design helps disentangle the key contributors to MethyNano’s performance gains, spanning both input features and architectural configurations.

All ablation experiments are performed on the *A. thaliana* dataset, and the results are summarized in Table 2. The unmodified model achieves the best overall performance across most metrics, indicating that both the integrated architecture and the full set of input features are important for robust 5mC prediction. In particular, masking the sequence input or the signal means leads to the most pronounced performance degradation, suggesting that sequence context and mean current signals provide highly informative cues for distinguishing methylated from unmethylated cytosines. In contrast, the signal-length features have a negligible effect on performance and can be considered largely non-essential under the current experimental setting. Notably, across all ablation conditions, the results consistently suggest that MethyNano’s multi-level feature extraction strategy and contrastive learning objective deliver clear advantages over conventional feature extraction and baseline fusion designs, supporting its effectiveness in modeling complex nanopore-derived biological signals.

### 3.5 Error characterization

To identify potential sources of systematic errors during inference, we perform a fine-grained analysis of misclassified samples (Fig. S6, available as supplementary data at *Bioinformatics* online). At the sequence level, we enumerate the top 20 most frequent motifs appearing in false positives (FP) and false negatives (FN) (Fig. S6a, available as supplementary data at *Bioinformatics* online). The results indicate that the model is more prone to misclassification under specific sequence contexts. For instance, motifs such as GCCAGGCTGGTCT and CTGCCTCAGCCTC appear in both the FP and FN lists, suggesting that these contexts may correspond to signal states with reduced separability thus blurred decision boundaries. At the signal level, we further introduce a motif-matched control analysis. For each FP sample, we select true negatives (TN) with the same motif among correctly predicted negative samples; similarly, for each FN sample, we select true positives (TP) sharing the same motif. Based on these matched controls, we compare the group-level characteristics of the normalized current waveforms at the central cytosine position (Fig. S6b, available as supplementary data at *Bioinformatics* online). We observe that under identical motif contexts, the erroneous group and its corresponding correct control exhibit highly similar global waveform profiles, with differences primarily manifesting as localized perturbations rather than substantial shape changes. We then project the central-position signal statistics (means and standard deviations) into a 2D space (Fig. S6c, available as supplementary data at *Bioinformatics* online), where FP samples largely overlap with their TN controls, and FN samples show no systematic shift relative to their matched TP controls. Collectively, these observations suggest that misclassifications are unlikely to be driven by single-position information at the target cytosine, but may instead arise from differences in cooperative, cross-position signal patterns.

## 4 Discussion

In this study, we propose MethyNano, a deep learning framework for nanopore 5mC detection. By integrating contrastive learning objectives (InfoNCE and SupCon), MethyNano accurately detects 5mC in the genomes of *A. thaliana*, *O. sativa*, and *H. sapiens*. On benchmark datasets, MethyNano achieves superior performance across all three species. In *A. thaliana*, it achieves a precision of 0.8426, a recall of 0.8321, an F1 score of 0.8366, an AUROC of 0.9202, and an AUPRC of 0.9215. It outperforms the strongest baseline, DeepPlant, by 0.65% in F1 score, 0.53% in AUROC, and 0.50% in AUPRC. Comparable improvements are observed in *H. sapiens* and *O. sativa*, where MethyNano leads on most metrics, although DeepPlant achieves higher precision. We further evaluate MethyNano’s generalization and robustness through cross-species and cross-motif experiments. Performance decreases under domain shift but MethyNano remains the top performer compared to all baselines. It provides reliable methylation predictions across diverse species and motif backgrounds. Moreover, our ablation studies reveal the decisive impact of both input design and architectural choices on MethyNano’s representational capacity. In particular, removing motif or signal means reduces F1 score by 7.2% and 7.6% compared to the full model. Among architectural components, replacing symmetric cross-attention with simple concatenation (mask_fuser) reduces F1 to 0.8250, a 1.4% drop. Disabling contrastive learning (mask_contrastive) lowers F1 to 0.8271, a 1.1% drop. Replacing multi-head CNN with standard convolution (mask_multiCNN) reduces F1 to 0.8291, a 0.8% drop. These results demonstrate that MethyNano’s multimodal fusion mechanism and contrastive learning strategy outperform conventional feature concatenation and basic architectural designs. To further assess practical usability, we benchmarked the methylation calling workflow of MethyNano and the baseline tools on 100 000 reads. The resulting wall clock time, peak RAM usage, and peak GPU memory usage are summarized in Table S7, available as supplementary data at *Bioinformatics* online. Finally, our systematic error analysis reveals that misclassifications primarily arise from complex inter-site signal patterns in specific sequence contexts, offering actionable directions for future model refinement.

Overall, MethyNano constitutes a powerful and robust tool for 5mC detection. Through multimodal fusion and contrastive representation learning, our model achieves improved robustness and higher predictive performance. As datasets expand and model designs continue to evolve, MethyNano has the potential to become an important tool for advancing epigenomic studies and downstream applications.

## Supplementary Material

btag348_Supplementary_Data

## References

[btag348-B1] Alagna N , Mündnich S, Miedema J et al ModiDeC: a multi-RNA modification classifier for direct nanopore sequencing. Nucleic Acids Res 2025;53:gkaf673. 40682823 10.1093/nar/gkaf673 PMC12276011

[btag348-B2] Arand J , Spieler D, Karius T et al In vivo control of CpG and non-CpG DNA methylation by DNA methyltransferases. PLoS Genet 2012;8:e1002750. 22761581 10.1371/journal.pgen.1002750 PMC3386304

[btag348-B3] Bai X , Yao H-C, Wu B et al DeepBAM: a high-accuracy single-molecule CpG methylation detection tool for Oxford nanopore sequencing. Brief Bioinform 2024;25:bbae413. 39177264 10.1093/bib/bbae413 PMC11342253

[btag348-B4] Bonet J , Chen M, Dabad M et al DeepMP: a deep learning tool to detect DNA base modifications on Nanopore sequencing data. Bioinformatics 2022;38:1235–43. 34718417 10.1093/bioinformatics/btab745 PMC8826383

[btag348-B5] Chan A , Naarmann-de Vries IS, Dieterich C. Ψ-co-mAFiA: concurrent detection of pseudouridine and m6A in single RNA molecules. Bioinformatics 2025;41:btaf536. 41002271 10.1093/bioinformatics/btaf536 PMC12552086

[btag348-B6] Chen H-X , Liu Z-D, Bai X et al Accurate cross-species 5mC detection for Oxford Nanopore sequencing in plants with DeepPlant. Nat Commun 2025;16:3227. 40185832 10.1038/s41467-025-58576-x PMC11971355

[btag348-B7] Cokus SJ , Feng S, Zhang X et al Shotgun bisulphite sequencing of the Arabidopsis genome reveals DNA methylation patterning. Nature 2008;452:215–9. 18278030 10.1038/nature06745 PMC2377394

[btag348-B8] Cruciani S , Delgado-Tejedor A, Pryszcz LP et al De novo basecalling of RNA modifications at single molecule and nucleotide resolution. Genome Biol 2025;26:38. 40001217 10.1186/s13059-025-03498-6 PMC11853310

[btag348-B9] Ehrlich M , Wang RY. 5-Methylcytosine in eukaryotic DNA. Science 1981;212:1350–7. 6262918 10.1126/science.6262918

[btag348-B10] ENCODE Project Consortium. An integrated encyclopedia of DNA elements in the human genome. Nature 2012;489:57. 22955616 10.1038/nature11247 PMC3439153

[btag348-B11] Flusberg BA , Webster DR, Lee JH et al Direct detection of DNA methylation during single-molecule, real-time sequencing. Nat Methods 2010;7:461–5. 20453866 10.1038/nmeth.1459 PMC2879396

[btag348-B12] Garalde DR , Snell EA, Jachimowicz D et al Highly parallel direct RNA sequencing on an array of nanopores. Nat Methods 2018;15:201–6. 29334379 10.1038/nmeth.4577

[btag348-B13] Genner R , Akeson S, Meredith M et al; CARD-long-read Team. Assessing DNA methylation detection for primary human tissue using Nanopore sequencing. Genome Res 2025;35:632–43. 40054862 10.1101/gr.279159.124 PMC12047266

[btag348-B14] Hadsell R , Chopra S, LeCun Y. Dimensionality reduction by learning an invariant mapping. In: *2006 IEEE Computer Society Conference on Computer Vision and Pattern Recognition (CVPR),* New York, NY, USA. Vol. 2. Los Alamitos, CA, USA: IEEE Computer Society, 2006, 1735–42.

[btag348-B15] He K, Zhang X, Ren S et al Deep residual learning for image recognition. In: *2016 IEEE Conference on Computer Vision and Pattern Recognition (CVPR)*, Las Vegas, NV, USA: IEEE, 2016, 770–8.

[btag348-B16] Howe KL , Contreras-Moreira B, De Silva N et al Ensembl Genomes 2020—enabling non-vertebrate genomic research. Nucleic Acids Res 2020;48:D689–95. 31598706 10.1093/nar/gkz890 PMC6943047

[btag348-B17] Hu H , Wang X, Zhang Y et al A comprehensive survey on contrastive learning. Neurocomputing (Amst) 2024;610:128645.

[btag348-B18] Huang S , Wylder AC, Pan T. Simultaneous nanopore profiling of mRNA m6A and pseudouridine reveals translation coordination. Nat Biotechnol 2024;42:1831–5. 38321115 10.1038/s41587-024-02135-0 PMC11300707

[btag348-B19] Jia Y , Gao B, Tan J et al Deep contrastive learning enables genome-wide virtual screening. Science 2026;391:eads9530. 41505557 10.1126/science.ads9530

[btag348-B20] Jones PA. Functions of DNA methylation: islands, start sites, gene bodies and beyond. Nat Rev Genet 2012;13:484–92. 22641018 10.1038/nrg3230

[btag348-B21] Jones PA , Takai D. The role of DNA methylation in mammalian epigenetics. Science 2001;293:1068–70. 11498573 10.1126/science.1063852

[btag348-B22] Kang G , Hwang H, Jeon H et al Comprehensive discovery of m6A sites in the human transcriptome at single-molecule resolution. Nat Commun 2025;17:664. 41398146 10.1038/s41467-025-67417-w PMC12816753

[btag348-B23] Khosla P, Teterwak P, Wang C et al Supervised contrastive learning. Adv Neural Inf Process Syst 2020;33:18661–73.

[btag348-B24] Krueger F , Andrews SR. Bismark: a flexible aligner and methylation caller for Bisulfite-Seq applications. Bioinformatics 2011;27:1571–2. 21493656 10.1093/bioinformatics/btr167 PMC3102221

[btag348-B25] Laszlo AH , Derrington IM, Brinkerhoff H et al Detection and mapping of 5-methylcytosine and 5-hydroxymethylcytosine with nanopore MspA. Proc Natl Acad Sci USA 2013;110:18904–9. 24167255 10.1073/pnas.1310240110 PMC3839702

[btag348-B26] Lister R , O’Malley RC, Tonti-Filippini J et al Highly integrated single-base resolution maps of the epigenome in Arabidopsis. Cell 2008;133:523–36. 18423832 10.1016/j.cell.2008.03.029 PMC2723732

[btag348-B27] McIntyre ABR , Alexander N, Grigorev K et al Single-molecule sequencing detection of N6 methyladenine in microbial reference materials. Nat Commun 2019;10:579. 30718479 10.1038/s41467-019-08289-9 PMC6362088

[btag348-B28] Ni P , Huang N, Zhang Z et al DeepSignal: detecting DNA methylation state from Nanopore sequencing reads using deep-learning. Bioinformatics 2019;35:4586–95. 30994904 10.1093/bioinformatics/btz276

[btag348-B29] Ni P , Huang N, Nie F et al Genome-wide detection of cytosine methylations in plant from Nanopore data using deep learning. Nat Commun 2021;12:5976. 34645826 10.1038/s41467-021-26278-9 PMC8514461

[btag348-B30] Niederhuth CE , Bewick AJ, Ji L et al Widespread natural variation of DNA methylation within angiosperms. Genome Biol 2016;17:194. 27671052 10.1186/s13059-016-1059-0 PMC5037628

[btag348-B31] Pagès-Gallego M , van Soest DMK, Besselink NJM et al Direct detection of 8-oxo-dG using nanopore sequencing. Nat Commun 2025;16:5236. 40473638 10.1038/s41467-025-60391-3 PMC12141616

[btag348-B32] Pérez E, Strub F, de Vries H et al FiLM: visual reasoning with a general conditioning layer. In: *Proceedings of the AAAI Conference on Artificial Intelligence*, New Orleans, LA, USA. Vol. 32, No. 1. Palo Alto, CA, USA: AAAI Press, 2018.

[btag348-B33] Perez M , Kimoto M, Rajakumar P et al Direct high-throughput deconvolution of non-canonical bases via nanopore sequencing and bootstrapped learning. Nat Commun 2025;16:6980. 40739090 10.1038/s41467-025-62347-z PMC12311055

[btag348-B34] Rand AC , Jain M, Eizenga JM et al Mapping DNA methylation with high-throughput nanopore sequencing. Nat Methods 2017;14:411–3. 28218897 10.1038/nmeth.4189 PMC5704956

[btag348-B35] Richards EJ. Natural epigenetic variation in plant species: a view from the field. Curr Opin Plant Biol 2011;14:204–9. 21478048 10.1016/j.pbi.2011.03.009

[btag348-B36] Simpson JT , Workman RE, Zuzarte PC et al Detecting DNA cytosine methylation using nanopore sequencing. Nat Methods 2017;14:407–10. 28218898 10.1038/nmeth.4184

[btag348-B37] Stoiber MH et al De novo identification of DNA modifications enabled by genome-guided nanopore signal processing. bioRxiv, 10.1101/094672, 2017, preprint: not peer reviewed.

[btag348-B38] van den Oord A , Li Y, Vinyals O. Representation learning with contrastive predictive coding. arXiv, 10.48550/arXiv.1807.03748, 2018, preprint: not peer reviewed.

[btag348-B39] Vaswani A, Shazeer N, Parmar N et al Attention is all you need. Adv Neural Inf Process Syst 2017;30:5998–6008.

[btag348-B40] Wallace EVB , Stoddart D, Heron AJ et al Identification of epigenetic DNA modifications with a protein nanopore. Chem Commun (Camb) 2010;46:8195–7. 20927439 10.1039/c0cc02864a PMC3147113

[btag348-B41] Wan YK , Hendra C, Pratanwanich PN et al Beyond sequencing: machine learning algorithms extract biology hidden in Nanopore signal data. Trends Genet 2022;38:246–57. 34711425 10.1016/j.tig.2021.09.001

[btag348-B42] Wheeler DL , Barrett T, Benson DA et al Database resources of the National Center for Biotechnology Information. Nucleic Acids Res 2008;36:D13–21. 18045790 10.1093/nar/gkm1000 PMC2238880

[btag348-B43] Xu L , Seki M. Recent advances in the detection of base modifications using the nanopore sequencer. J Hum Genet 2020;65:25–33. 31602005 10.1038/s10038-019-0679-0 PMC7087776

[btag348-B44] Yin C , Wang R, Qiao J et al NanoCon: contrastive learning-based deep hybrid network for nanopore methylation detection. Bioinformatics 2024;40:btae046. 38305428 10.1093/bioinformatics/btae046 PMC10873575

[btag348-B45] Yuen ZW-S , Srivastava A, Daniel R et al Systematic benchmarking of tools for CpG methylation detection from nanopore sequencing. Nat Commun 2021;12:3438. 34103501 10.1038/s41467-021-23778-6 PMC8187371

[btag348-B46] Zhang C , Wu R, Sun F et al Parallel molecular data storage by printing epigenetic bits on DNA. Nature 2024;634:824–32. 39443776 10.1038/s41586-024-08040-5 PMC11499255

[btag348-B47] Zhang Y , Wu Y, Ma J et al DirectRM: integrated detection of landscape and crosstalk between multiple RNA modifications using direct RNA sequencing. Nat Commun 2025;16:9450. 41145497 10.1038/s41467-025-64495-8 PMC12559715

[btag348-B48] Zhang YZ, Yamaguchi K, Hatakeyama S et al On the application of BERT models for nanopore methylation detection. In: *2021 IEEE International Conference on Bioinformatics and Biomedicine (BIBM)*, Houston: IEEE; 2021, 320–7.

