# Supplementary material for: MethyNano: supervised contrastive pretraining enables robust and generalizable methylation detection from nanopore sequencing
Source: Bioinformatics. 2026 May 28;42(6):btag348. doi: 10.1093/bioinformatics/btag348 (PMC13296999; doi:10.1093/bioinformatics/btag348)
Supplement: btag348_Supplementary_Data [file btag348_supplementary_data.zip › supplementary materials.docx]

**Supplemental Materials for**

**MethyNano: supervised contrastive pretraining enables robust and generalizable methylation detection from nanopore sequencing**

*Jiahui Yan^1^, Yujie Chen^1^, Yucong Gong ^2^, Cheng Zhang^2*^, Jing Yang^1*^*

^1^School of Control and Computer Engineering, North China Electric Power University, Beijing, 102206, China

^2^School of Computer Science, Key Laboratory of High Confidence Software Technologies, Peking University, Beijing, 100871, China

*** Correspondence:**

Jing Yang: yjzcdd_2000@ncepu.edu.cn


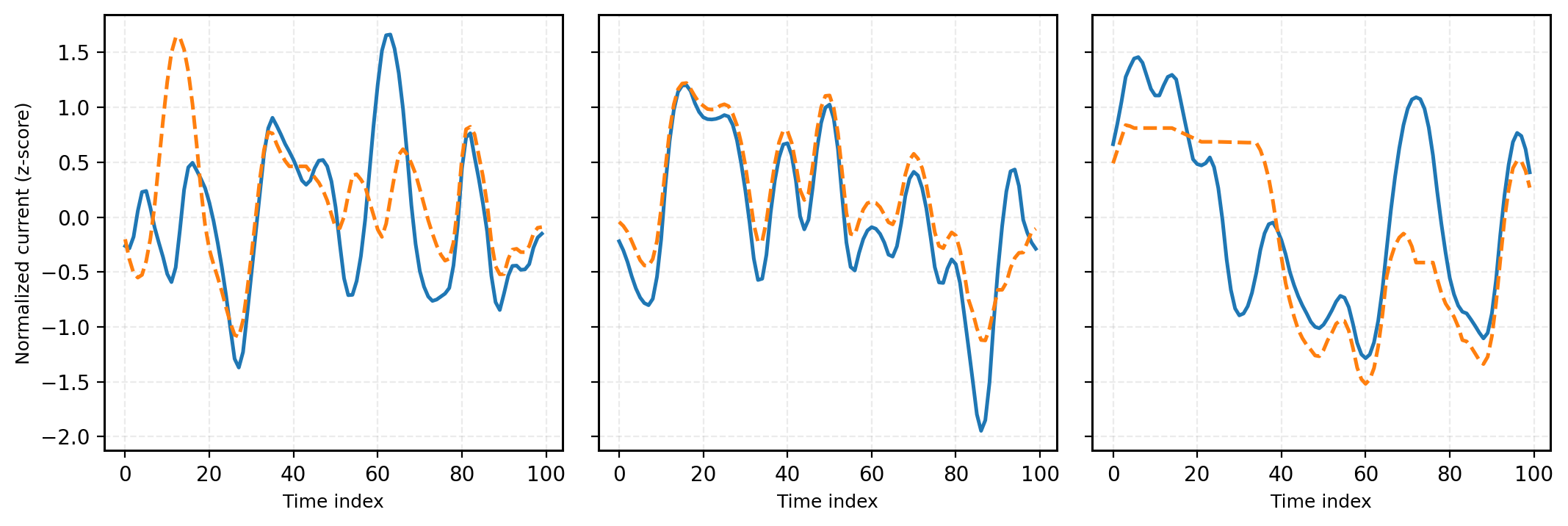

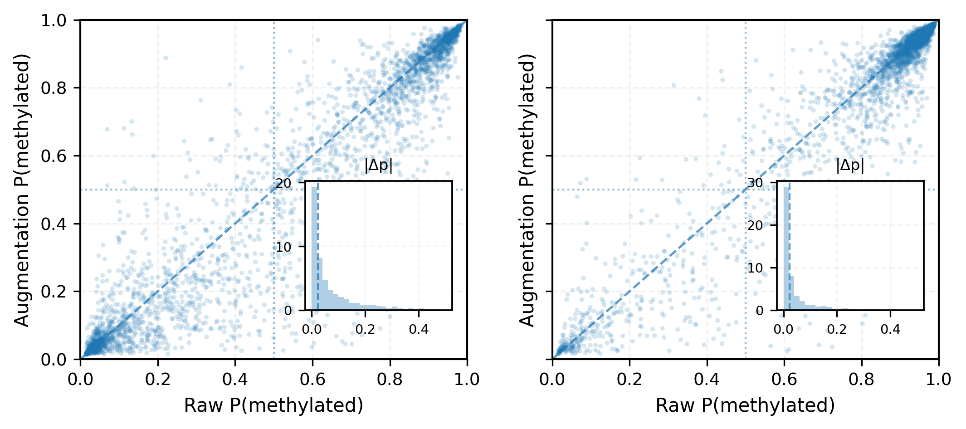

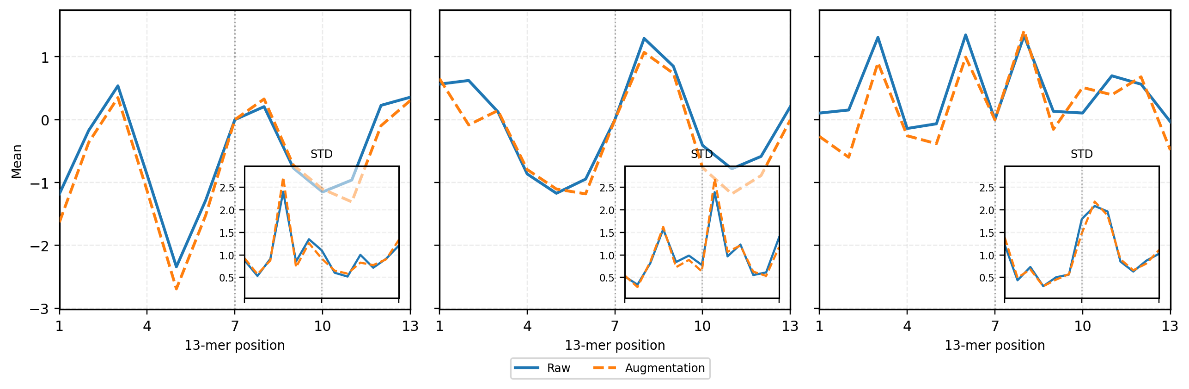


a

b

c

**Fig. S1** Consistency evaluation between raw signals and their augmented counterparts. **a.** Normalized current traces for several examples which aligned at the central target cytosine (C). The augmented traces closely match the raw signals in overall shape, with only localized variations introduced by augmentation. **b.** Event-level summary statistics across the 13-mer positions for several examples. The main panel shows the mean current at each position, while the inset reports the corresponding position-wise standard deviation (std). The overall profiles remain largely consistent before and after augmentation, suggesting that augmentation does not systematically distort the statistical structure of signal events. **c.** Scatter plot of model-predicted probabilities for augmented versus raw samples. The inset shows the distribution of $\left| \Delta p \right|=\left| p_{Augmentation}-p_{Raw} \right|$, which we use to quantify the prediction shift between paired raw and augmented samples. Most $|\Delta p|$ values are close to zero, and large deviations are rare.


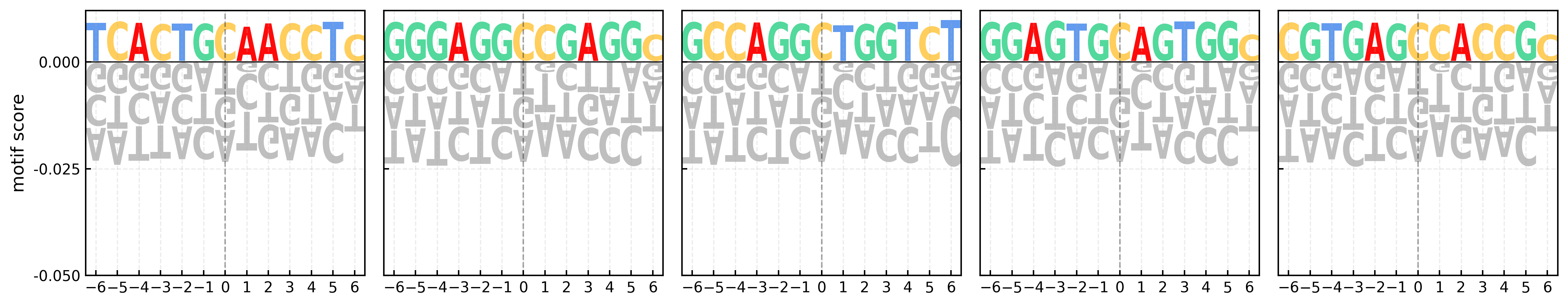

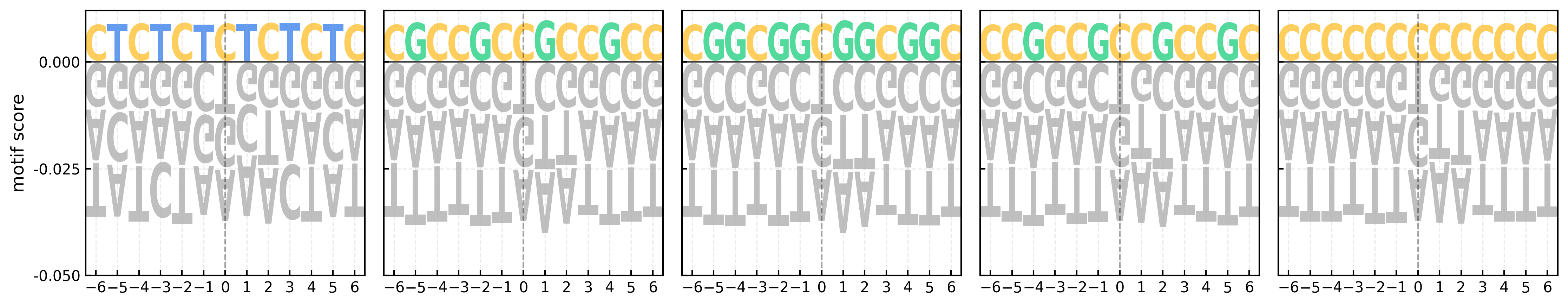

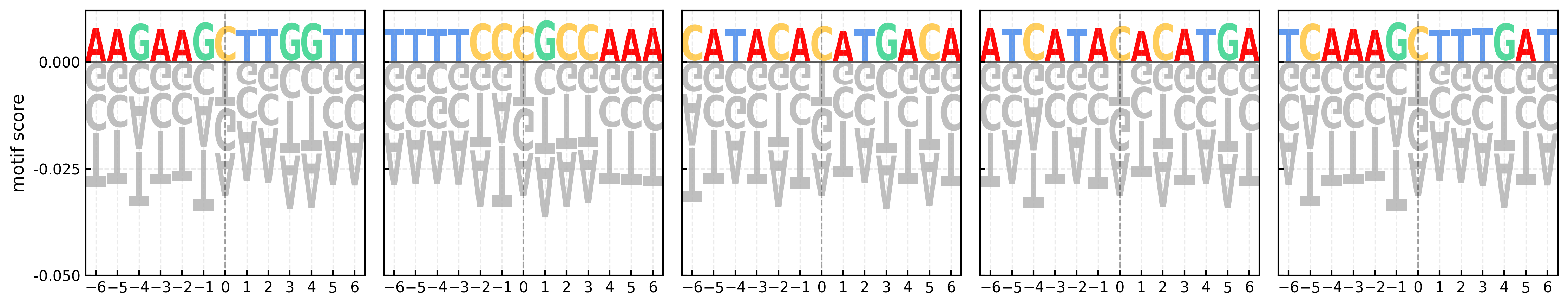


a

b

c

**Fig. S2** Visualization of the top five most frequent motifs in the datasets used in our study: **a.** *A. thaliana* (Bai *et al.* 2024), **b.** *O. sativa* (Bai *et al.* 2024), **c.** *H. sapiens* (Genner *et al.* 2025).

**Fig. S3** Cross-dataset generalization evaluation of models trained on *A. thaliana* (Wheeler DL, *et al.* 2007). (**a-e**) Performance curves of five models (MethyNano, NanoCon (Yin C *et al.* 2024), remora (Oxford Nanopore Technologies), rockfish (Stanojević D, *et al.* 2024), and DeepPlant (Chen HX, *et al.* 2025)) trained on *A. thaliana* and tested on three datasets (*A. thaliana*, *O. sativa*, and *H. sapiens*). AUROC, AUPRC, and F1 score curves are shown for each model. Curves are color-coded by test dataset, allowing a direct comparison of cross-species generalization performance.

e

d

c

b

a


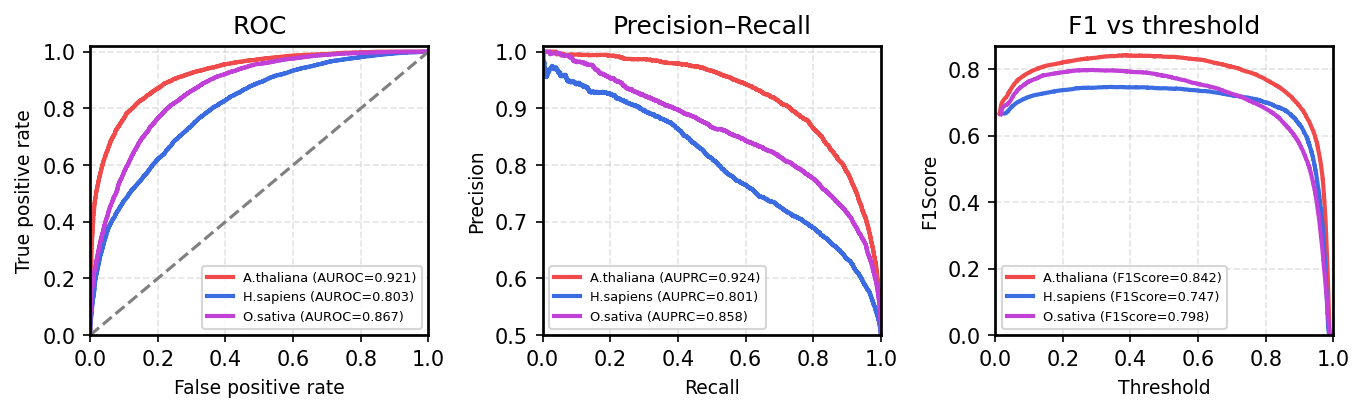

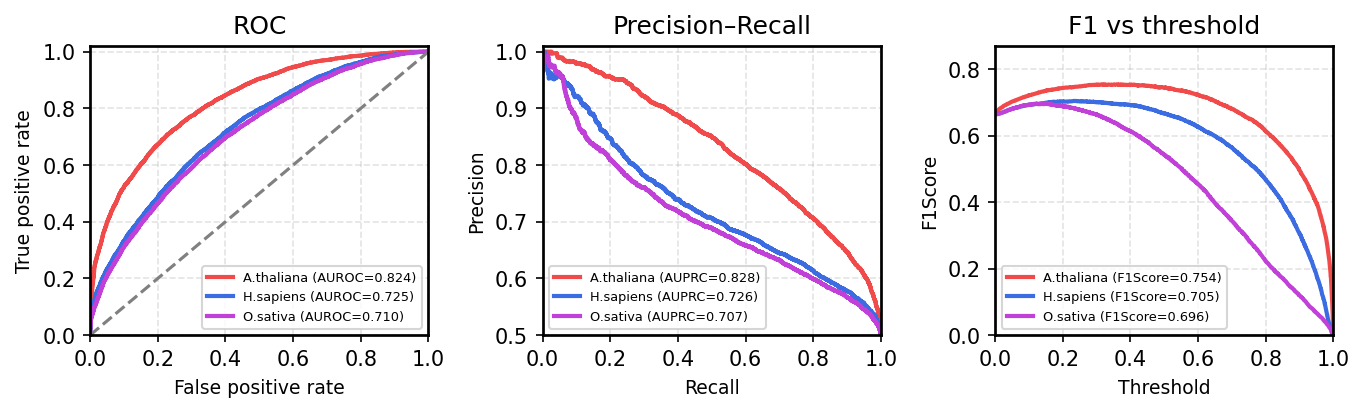

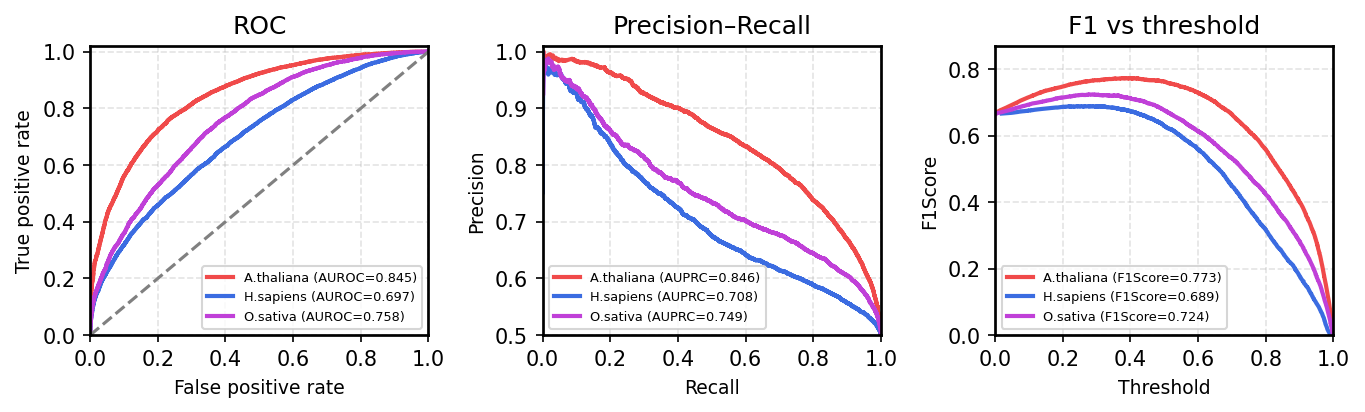

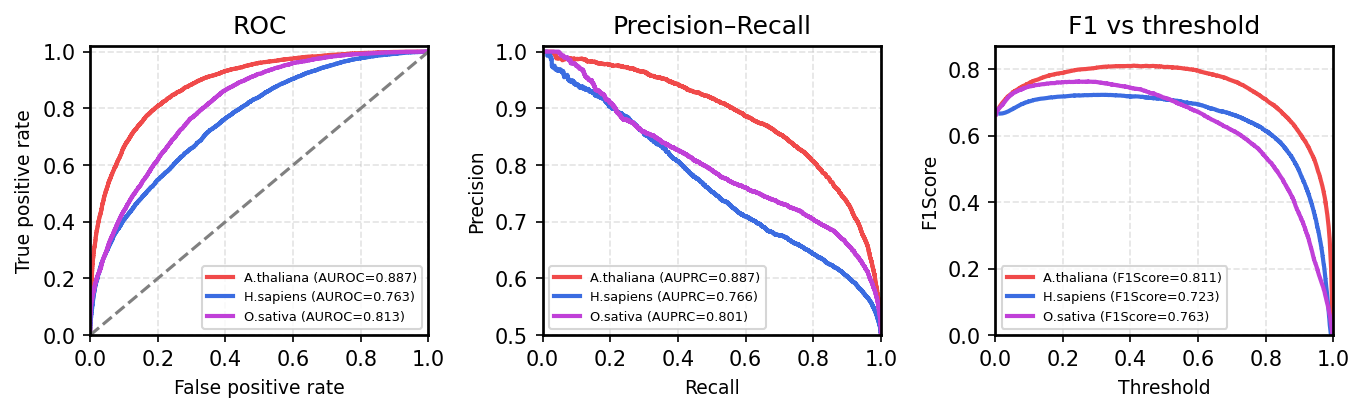

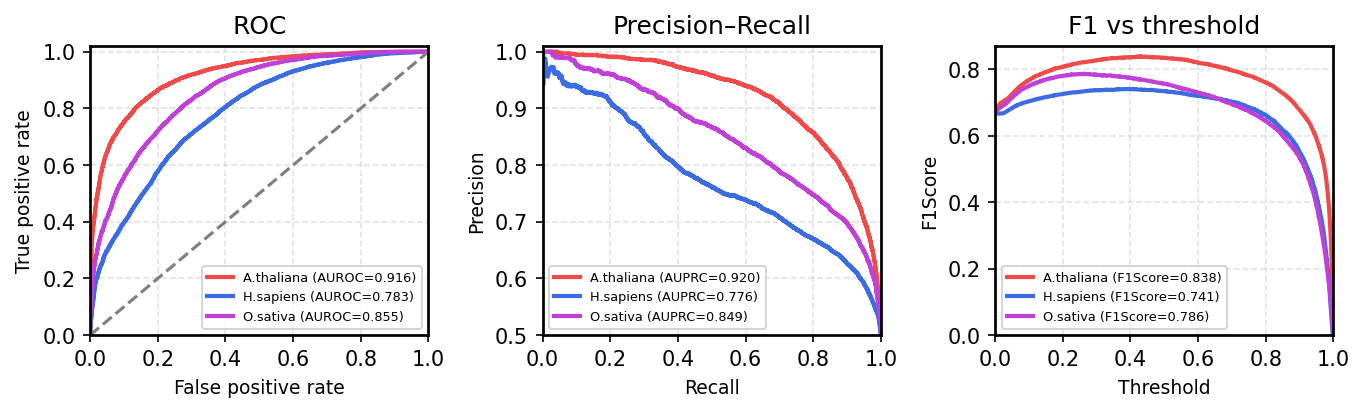

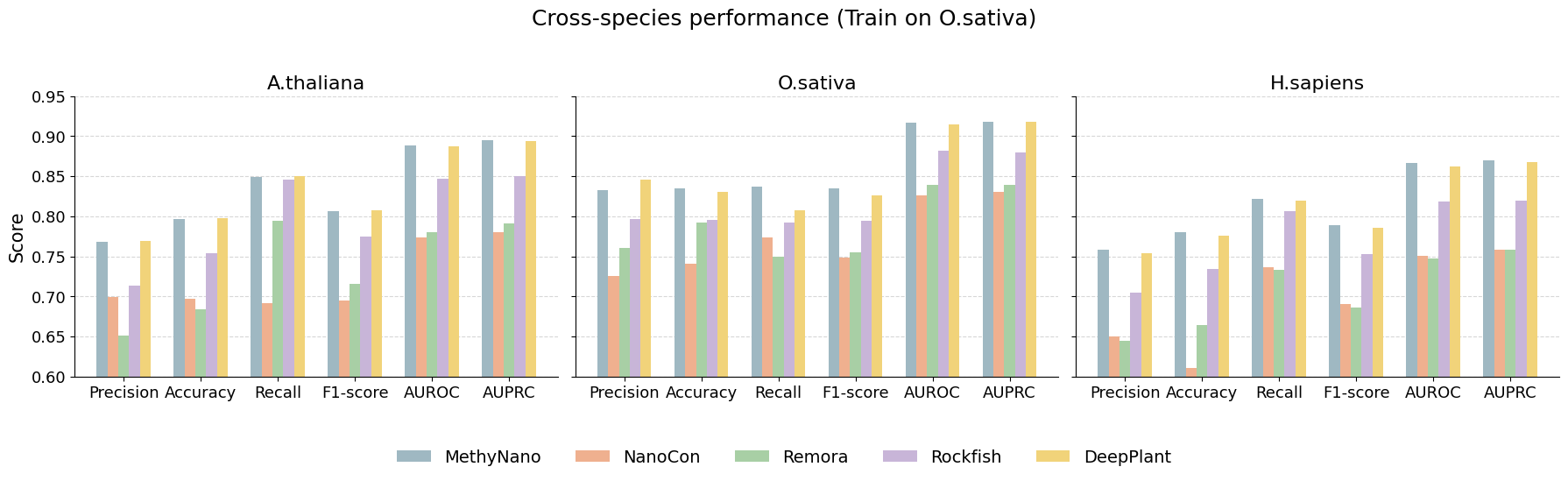

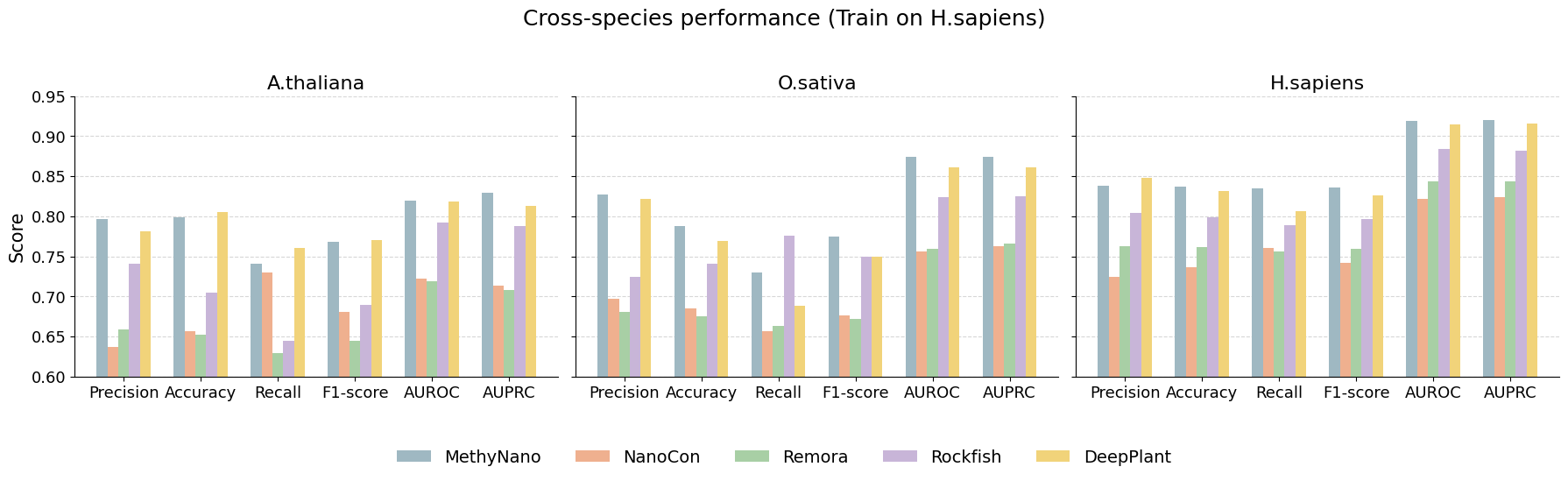


av

bv

**Fig. S4** Cross-species testing performance of models trained on the *O. sativa* and *H. sapiens* datasets.

**Fig. S5** Comparison of cross-motif generalization performance. This figure systematically evaluates the robustness and generalization ability of five models when transferred across different methylation motifs. Panels (**a–c**) correspond to training on CpG, CHG, and CHH, respectively. For each training motif, models are evaluated on test data from all three motifs using six metrics (Accuracy, Precision, Recall, F1 score, AUROC, and AUPRC). Overall, all models achieve their best performance when the training and testing motifs match. However, performance degradation under cross-motif transfer varies substantially across models. Notably, MethyNano consistently attains the best performance across all motif contexts.

c

b

a


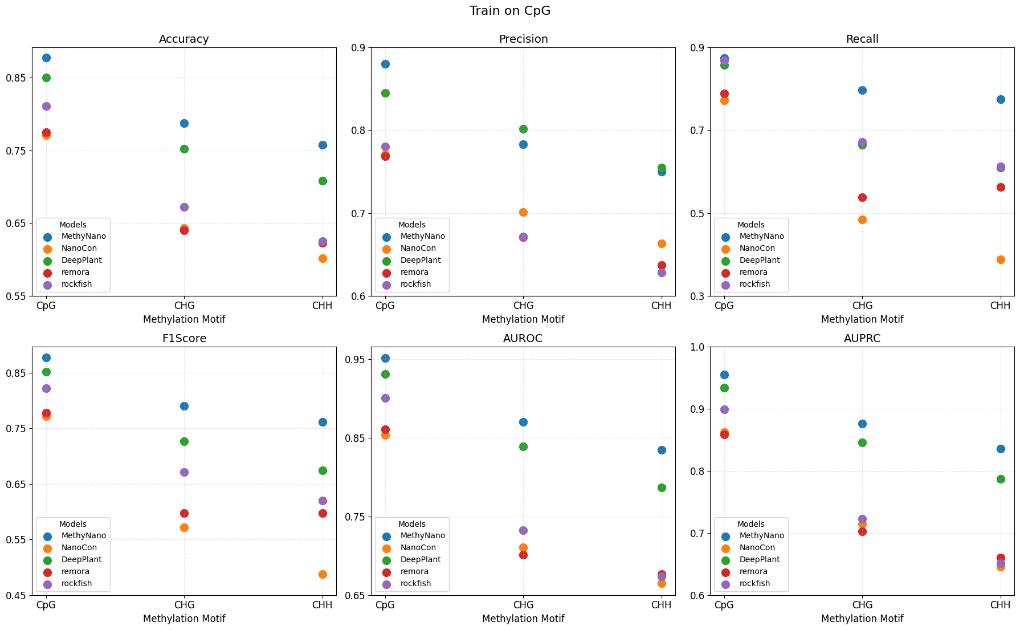

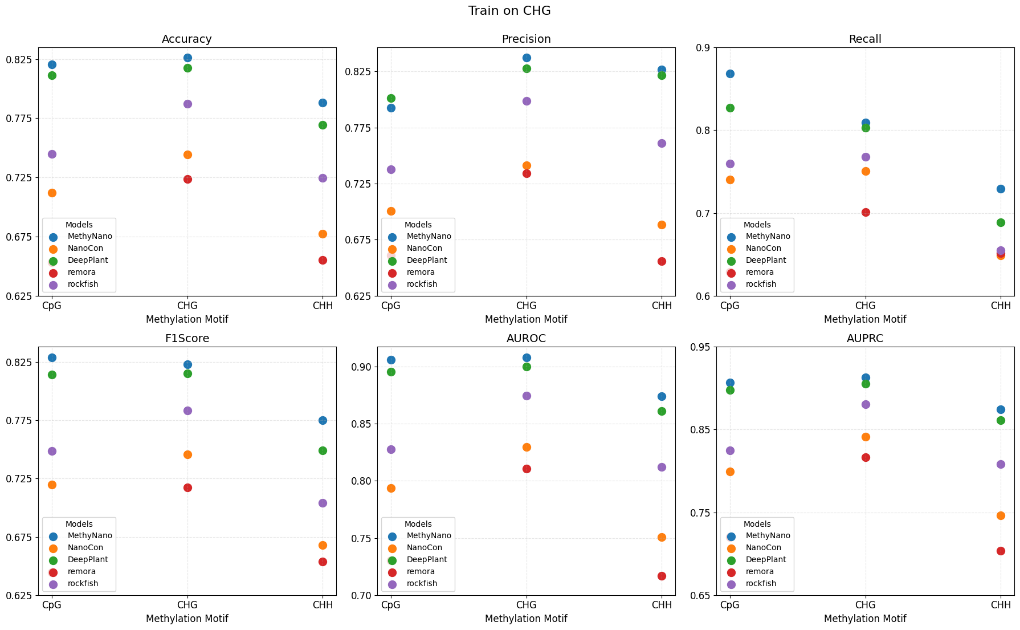

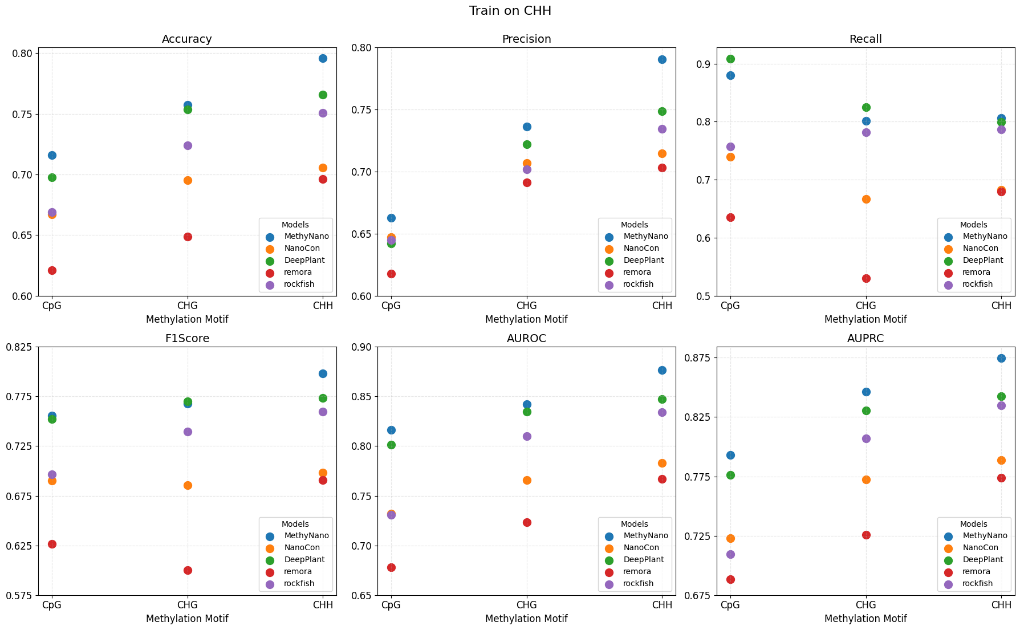

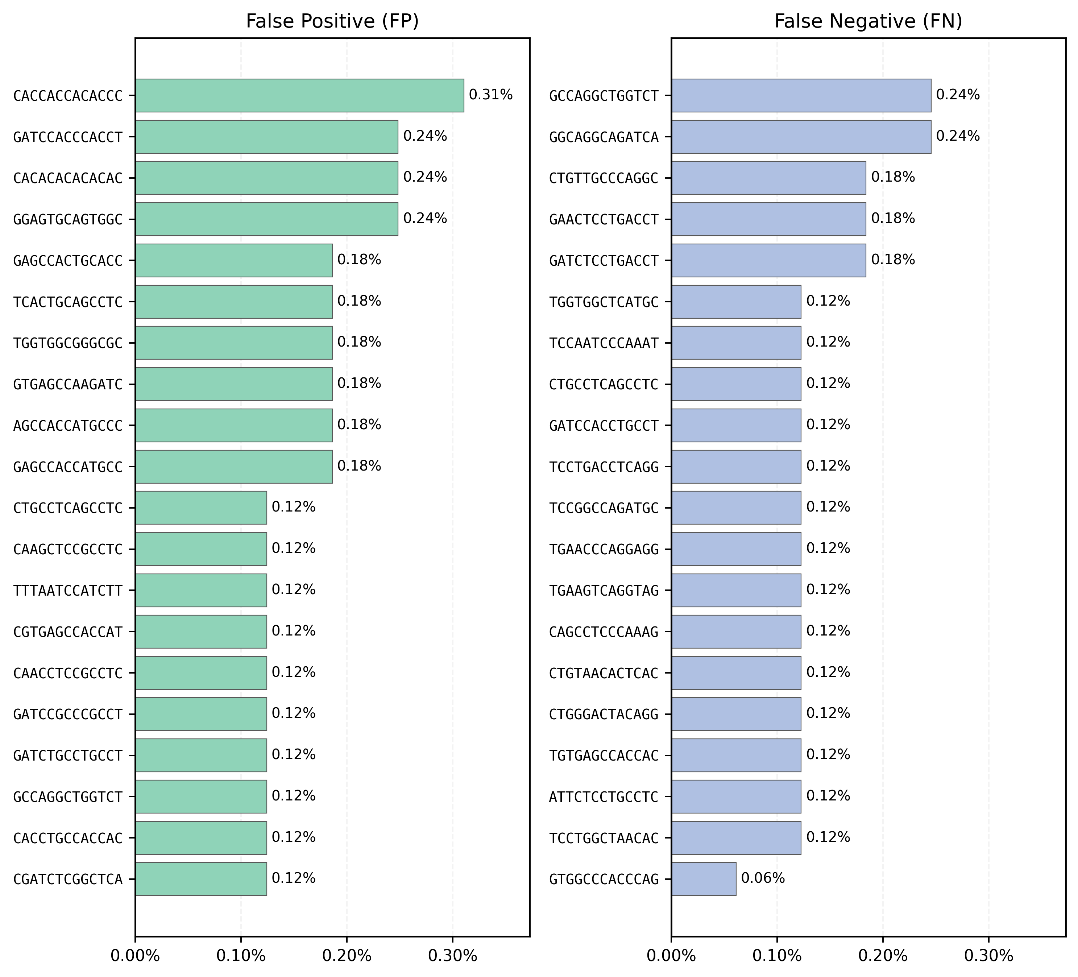

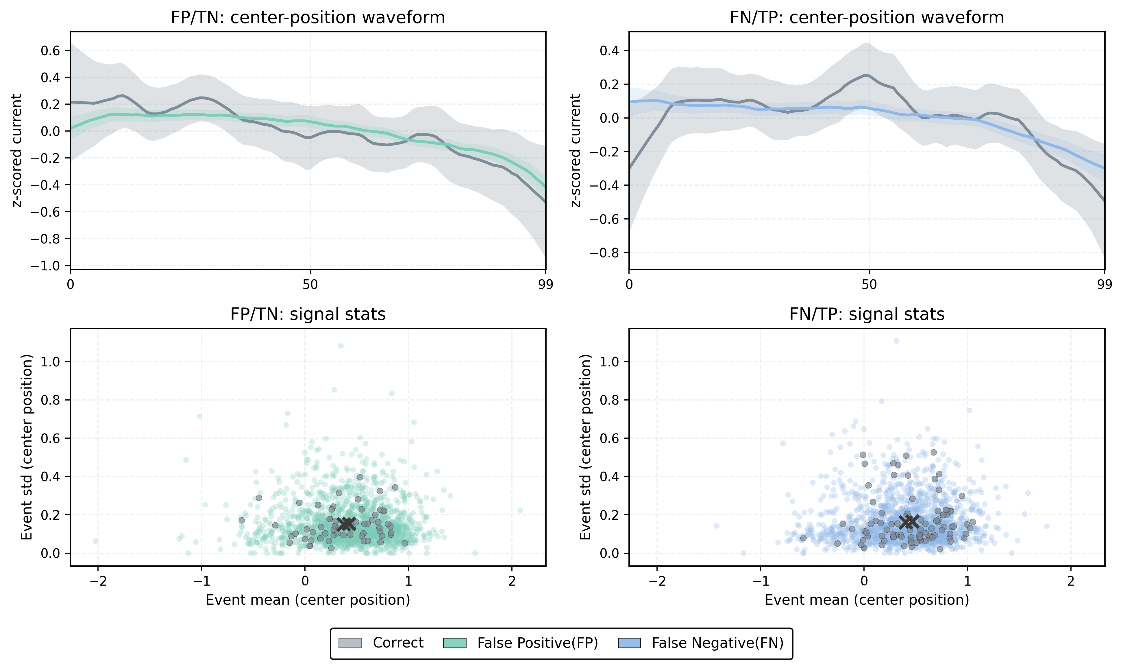


a

b

c

**Fig. S6** Analysis of sequence and current-derived statistical features in misclassified samples. **a.** A systematic analysis of error sources at the 13-mer sequence level. The left panel shows the top 20 sequences most frequently observed among false positives (FP), whereas the right panel shows the top 20 sequences most frequently observed among false negatives (FN). Notably, the motifs GCCAGGCTGGTCT and CTGCCTCAGCCTC appear in both lists, suggesting that the model has limited discriminability for these motifs. **b.** Group-level comparison of normalized current waveforms at the central cytosine (C) of the motif. The left plot contrasts FP samples with matched true negatives (TN) sharing the same 13-mer, while the right plot contrasts FN samples with matched true positives (TP). **c.** Two-dimensional distributions of current-derived statistics at the central position, with “×” marking the centroid (central tendency) of each group. Misclassified samples largely overlap with their correctly classified counterparts in the (mean, std) plane, and their centroids are close. This indicates that the mean and variance at the central position alone are insufficient to separate FP/FN from correctly classified samples. Instead, the model likely needs to incorporate cooperative effects from neighboring positions for more reliable discrimination.


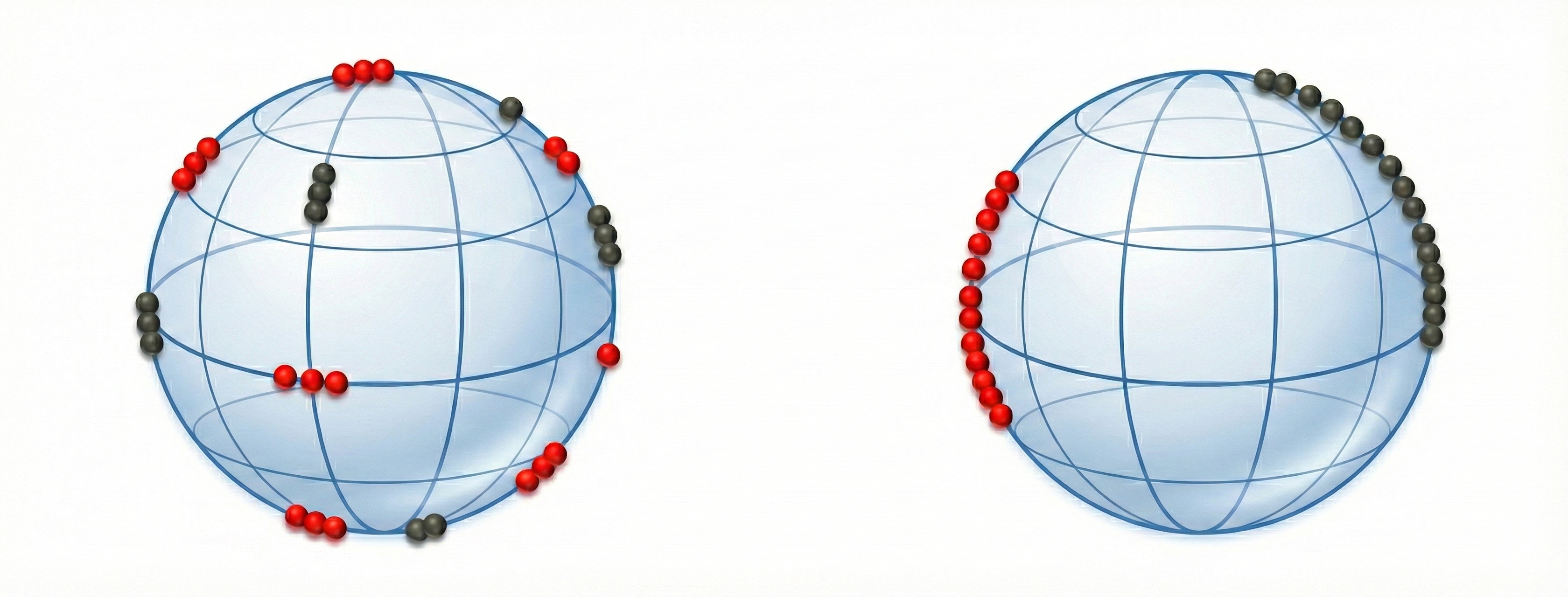


InfoNCE(Self Supervised Contrastive)

SupCon(Supervised Contrastive)

**Fig. S7** Visual comparison of InfoNCE and SupCon objectives. The left panel shows that InfoNCE emphasizes instance-level discrimination by pulling paired samples closer while pushing other samples apart, resulting in embeddings distributed over a hypersphere.The right panel shows that SupCon leverages label information to draw all samples from the same class together,thereby forming compact and well-separated class clusters in the embedding space.

**
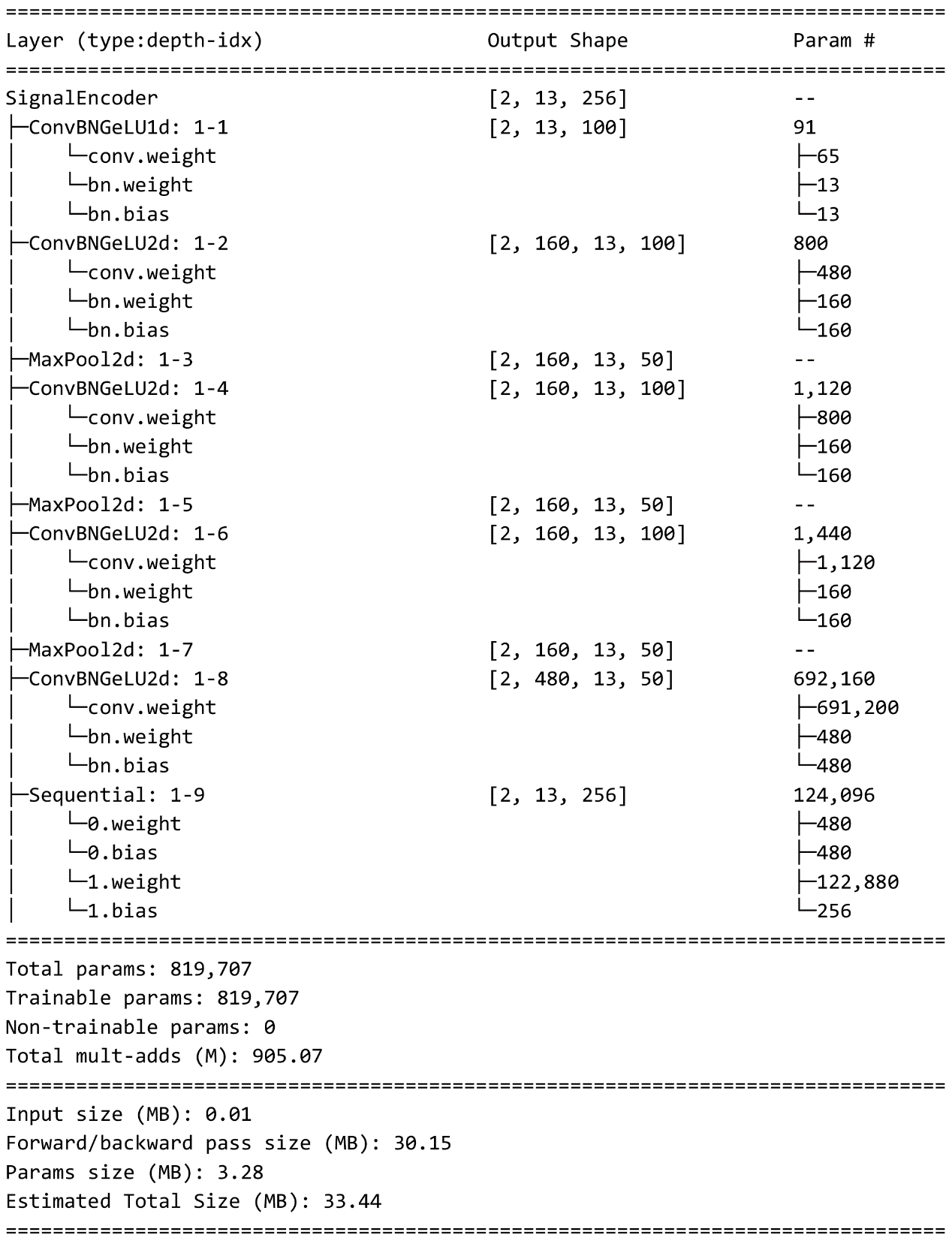
Table S1** Model architecture of MethyNano (Signal Encoder)

**
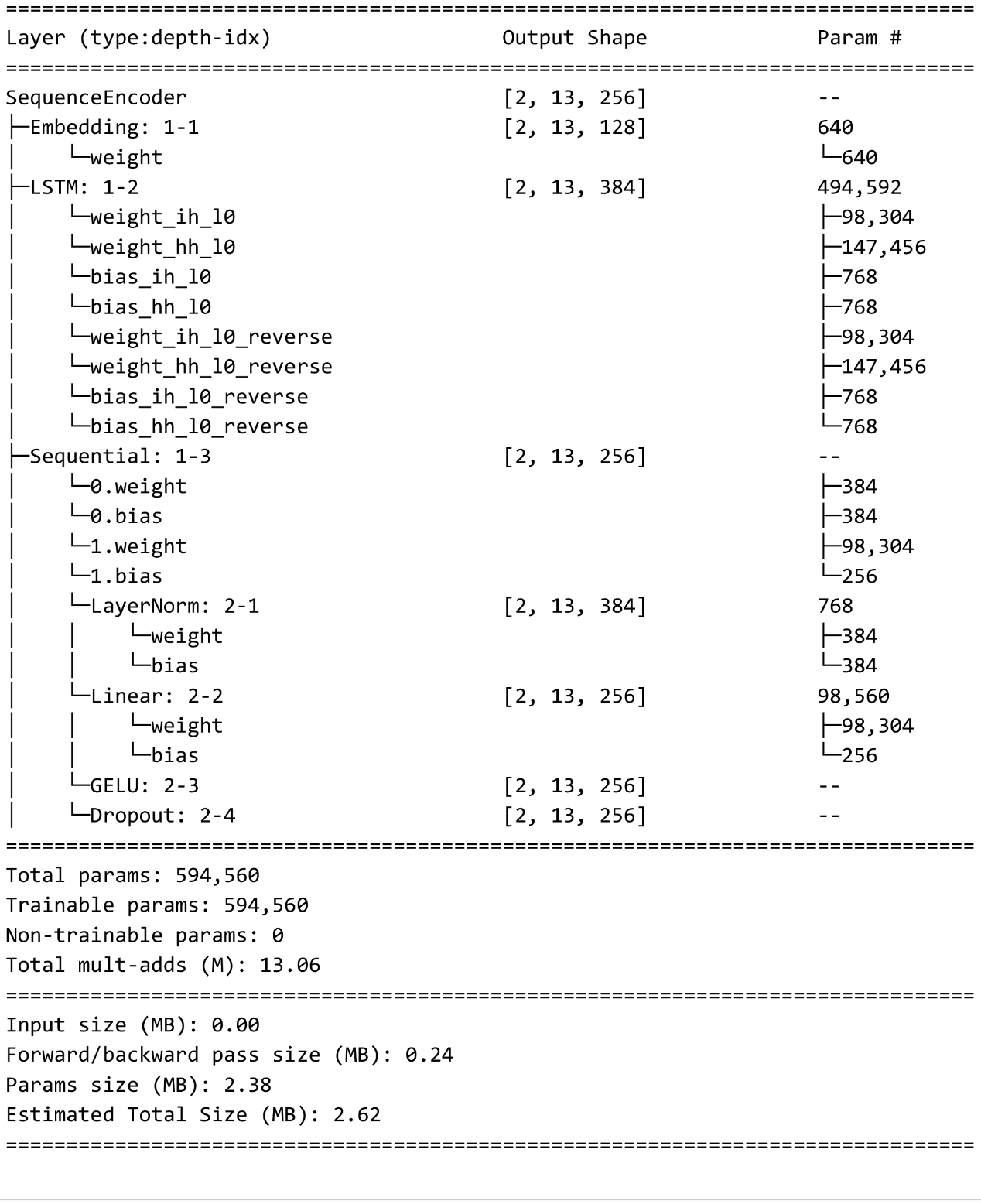
Table S2** Model architecture of MethyNano (Sequence Encoder)

**Table S3** Model architecture of MethyNano (Cross Modal Fuser)
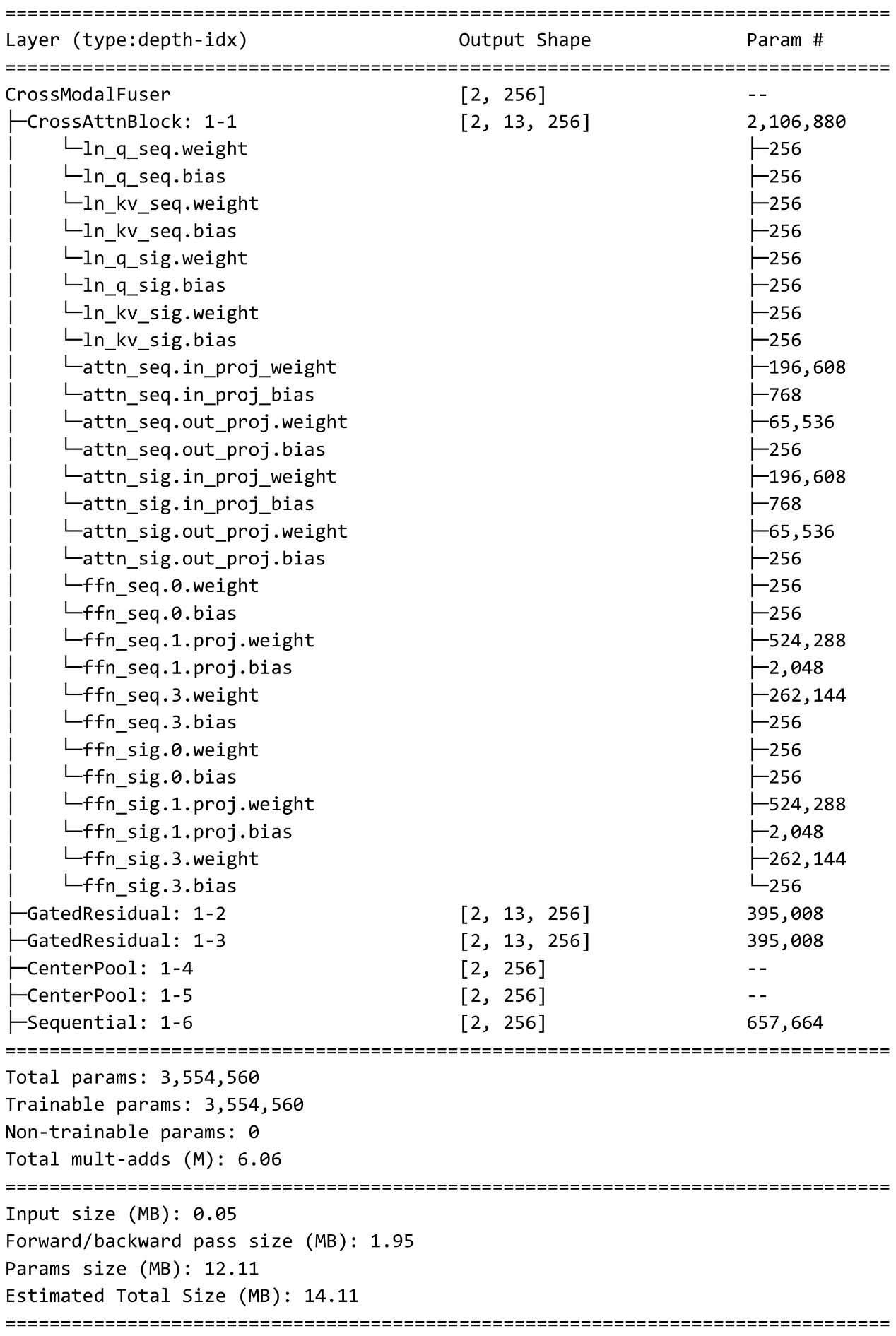


**Table S4** Model architecture of MethyNano (Projection Head)
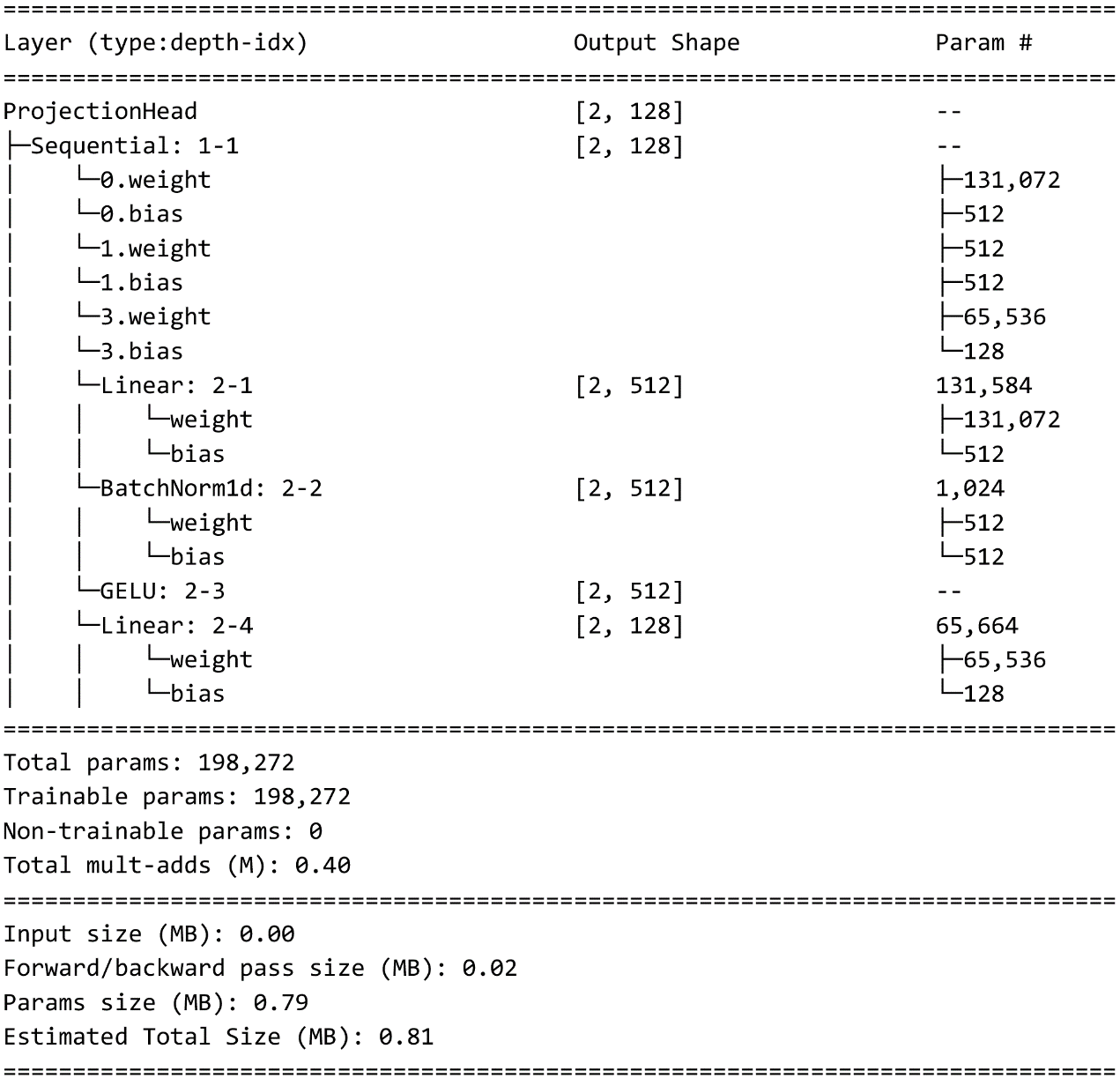


**
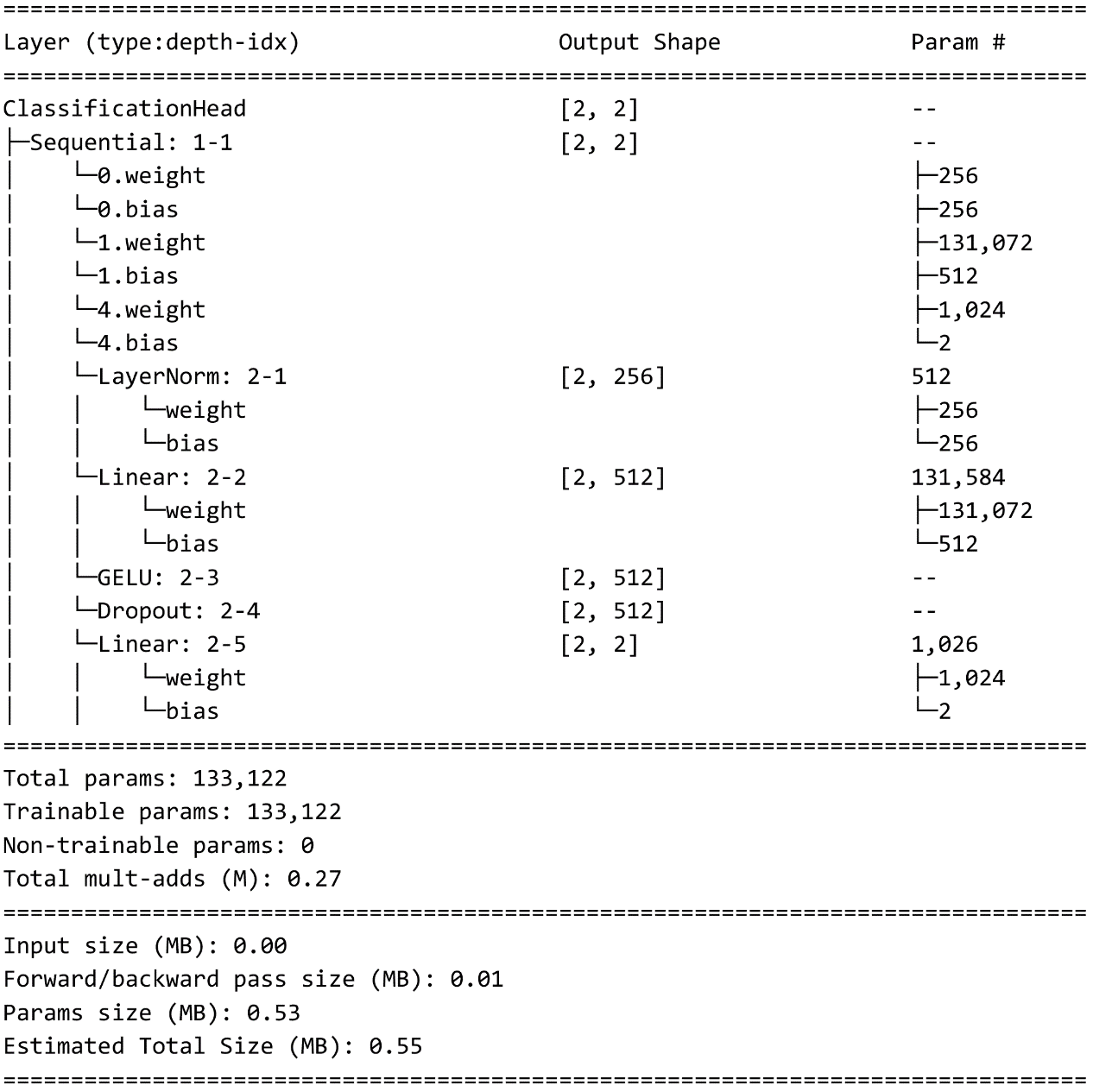
Table S5** Model architecture of MethyNano (Classification Head)

**Table S6** Distribution of CpG, CHG, and CHH samples across species-specific datasets.

| Dataset | CpG | CHG | CHH | TOTAL |
| --- | --- | --- | --- | --- |
| 1. *thaliana* | 5166 | 35330 | 159504 | 200000 |
| *O. sativa* | 8442 | 38505 | 153053 | 200000 |
| *H. sapiens* | 15400 | 49176 | 135434 | 200000 |

**Table S7** Runtime comparison among MethyNano and other tools. This table presents the execution time and resource usage of MethyNano and other tools on 100,000 reads. Benchmarking was conducted on a workstation equipped with an Intel Core i9-13900K CPU and an NVIDIA GeForce RTX 4090 GPU.

| model | Wall clock time (s) | Peak RAM usage (MB) | Peak GPU memory usage (MB) |
| --- | --- | --- | --- |
| MethyNano | 900.5 | 7228 | 2854 |
| rockfish | 10081.07 | 6900.06 | 384 |
| remora | 540.8 | 4091.94 | 1312 |
| DeepPlant | 470.67 | 3398.56 | 1634 |

**References**

Bai X, *et al.* DeepBAM: a high-accuracy single-molecule CpG methylation detection tool for Oxford nanopore sequencing. Brief Bioinform 2024;25: bbae413.

Chen HX, *et al.* Accurate cross-species 5mC detection for Oxford Nanopore sequencing in plants with DeepPlant. *Nat Commun* 2025; **16**:3227.

Genner R, *et al.* Assessing DNA methylation detection for primary human tissue using Nanopore sequencing. *Genome Res* 2025; **35**:632–43.

Stanojević D, *et al.* Rockfish: a transformer-based model for accurate 5-methylcytosine prediction from nanopore sequencing. *Nat Commun* 2024; **15**:5580.

Yin C *et al.* NanoCon: contrastive learning-based deep hybrid network for nanopore methylation detection. *Bioinformatics* 2024;**40**: btae046.
